# A numerical design framework for unloading excavations in deep potash mining

**DOI:** 10.1038/s41598-025-29177-x

**Published:** 2025-12-22

**Authors:** Yiqiang Zhang, Siarhei Lapatsin, Michael Zhuravkov, Guangbin Yu, Yiqian He, Pavel Piaredryi

**Affiliations:** 1 School of Mechatronics Engineering, Harbin Institute of Technology, Harbin, 150001 China; 2 Theoretical and Applied Mechanics Department, Belarusian State University, Minsk, 220030 Belarus; 3 Department of Engineering Mechanics, Dalian University of Technology, Dalian, 116023 China; 4 DUT-BSU Joint Institute, Dalian University of Technology, Dalian, 116023 China

**Keywords:** Unloading excavations, Deep potash mining, Safety measures, Finite element method, Limit state analysis, Complex limit state criterion, Salt rock mechanics, Engineering, Solid Earth sciences

## Abstract

The stability of deep underground structures in potash mining is challenged by significant rock pressure and the inhomogeneous structure of the enclosing rock mass, often requiring innovative support strategies beyond conventional methods. This study investigates the deliberate use of unloading excavations as a safety measure for underground structures in potash salt rock masses at depths ranging from 300 to 1200 m. Through an extensive series of finite element method (FEM) simulations incorporating an original complex limit state criterion, the effectiveness of this approach is evaluated by comparing the extent of limit state zones across the investigated depth range. The FEM model was indirectly validated against long-term field deformation data from a single mining excavation at the Starobin potash deposit, demonstrating its ability to capture the displacement field over a one-year period. Numerical results demonstrate that the strategic placement of unloading excavations can reduce the computed size of limit state zones by 7% to over 90% across the studied scenarios. For the conditions of the Starobin deposit, the analysis suggests design parameters including a 3-meter diameter for the unloading excavation and a placement distance of 1.5–7 m from the main excavation, with a distance of 5–6 m being the most significant in reducing limit state zones. The findings provide initial approximations and preliminary design guidelines, indicating that this technique can contribute to enhancing the stability of deep geotechnical structures in potash mining, while also pointing to its potential relevance for other geotechnical projects.

## Introduction

The safety and stability of underground geotechnical structures, including tunnels, mining excavations, chambers, caverns and underground storages remain critical concerns in modern engineering and mining practices^1,2^. These structures are integral to infrastructure development, resource extraction, and urban planning, yet their design and maintenance pose significant challenges due to the complex behavior of rock masses under external and internal loading. Over the years, a wide array of effective and well-verified methods, techniques, and solutions have been developed to address these challenges (see e.g^3,4^. However, despite these advancements, identifying the most efficient and reliable design approach for underground structures continues to be a pressing issue, particularly in the context of optimizing safety, stability, and cost-effectiveness^5,6^. This is especially relevant for deep mining, since classic safety measures are not always efficient at great depths^7–9^.

A central aspect of designing underground structures is the mitigation of rock pressure in the vicinity of these structures and the reinforcement of their outlines to prevent failure^10–12^. To achieve this, various safety measures are commonly employed. For example, such protection and securing measures as anchoring, rock bolts, various linings and compensational gaps are widely used^13–16^. These techniques aim to strengthen the excavations’ outline and enhance the structural integrity of underground excavations. In addition to these widely used methods there are also some other approaches to increase the safety and durability of underground structures^17,18^.

Among these, an alternative protection method called “unloading excavations” is used^17,19–21^. Unloading, as a concept, refers to the reduction of rock pressure at the outline of an excavation. This method involves creating additional excavations or a system of excavations near the designed (main) geotechnical structure. The purpose of these auxiliary (unloading) excavations is to concentrate rock pressure in their vicinity, thereby reducing the rock pressure near the main structure and improving its strength, stability and durability. Such unloading excavations are usually created before the main structures. The main effect of such excavations is a special redistribution of the stress-strain state in the enclosing rock mass near the designed excavations or other underground structures. The redistribution occurs in such a way that the rock mass in the vicinity of the unloading excavation absorbs the additional stress and deformation, which initially occurs in the unloading excavation region rather than near the main excavation. Note that in comparison with traditional methods like anchoring, rock bolts, linings and compensational gaps^13–16^ the unloading excavations do not require continuous maintenance, which makes this technique a more stable and long-term solution. Furthermore, the full collapse of such excavations is not hazardous to personnel, as they are not operational. In addition to that, the mechanics behind unloading excavations is fundamentally different from that behind traditional safety measures, since they aim to redistribute the stress field near openings and structures rather than directly strengthening the excavation outline. In this paper we focus on unloading excavations as a standalone safety measure for underground structures.

Over the decades, the technology of unloading excavations was implemented at potash mining deposits of the Republic of Belarus^19^. The theoretical basis for the use of unloading excavations was developed at the end of the 20th century^19^. However, preliminary experimental studies on the effectiveness of the unloading excavations at great depths (over 900 m) in potash mines failed to demonstrate positive results^19^. Consequently, the use of such excavations as safety measures for mining operations was restricted to moderate depths (300–900 m). This fact is taken into account in the geotechnical guideline for the Republic of Belarus^20^. However, these limited experimental studies were conducted decades ago and investigated only a few cases of the unloading excavations placement, since creating unloading excavations very close to the main structures is very risky and expensive at great depths. In this article we revise the possible use of unloading excavations at various depths using modern numerical simulation tools. This allows to investigate various parameters of excavation placement neglecting the risks and economic costs. The most crucial parameter investigated in the current study is the optimal distance between main and unloading excavations.

Although some publications exist on this topic, discussion of unloading excavations remains limited in the international research literature. Nevertheless, these excavations are particularly effective in environments with high rock pressure and stress concentrations, which pose significant risks to the stability of underground structures. For instance, in coal mining, unloading excavations have been used to mitigate rock bursts and roof collapses, significantly improving safety and operational efficiency^21^. Similarly, in deep tunneling, they have been employed to reduce the risk of tunnel convergence and lining failure in high-stress conditions^22^.

Furthermore, a body of research investigates the behavior of twin or multiple neighboring tunnels and the corresponding unloading effects^23–27^. For example, the article^23^ examines twin-tunnel interactions in slopes, demonstrating how optimized excavation geometry can improve stress redistribution and system stability. The work^24^ deals with near-surface twin-tunnel interaction in soft ground. The authors of^26^ focus specifically on the spacing between closely located tunnels, which aligns with a key goal of the present research. The work^27^ emphasizes the potential for asymmetric failure around multiple deep excavations, correlating with our findings. We separately highlight the recent work^28^ due to its object of investigation being very similar to our study in terms of both the parameters studied and the numerical approach adopted. We also cite works^29,30^ as they are focused on unloading effects in the rock mass. All the mentioned works are strategically aligned with the present study, as they utilize advanced numerical simulation tools to investigate rock mass behavior near twin excavations or the unloading effect. However, in all these cases, the unloading effect is examined only as an incidental byproduct of twin-tunnel construction, a necessity driven by engineering needs, rather than as a deliberate, standalone safety measure.

This article examines the standalone use of unloading excavations as a safety measure for underground structures in potash mining, including at great depths. We present a comparative numerical investigation supported by field data on excavation deformation from the Starobin potash deposit in the Republic of Belarus^31^. The proposed methodology combines extensive series of FEM simulations with the limit state concept, which allows us to move beyond the analysis of individual stress components towards a comprehensive evaluation of the rock mass’s proximity to failure. A complex limit state criterion, introduced in the authors’ previous, is applied for this purpose (see Sect. “Limit state calculation method”). Consequently, this study provides a methodological framework for optimizing the design of unloading excavations, suggesting a strategy to potentially enhance the safety and stability of underground excavations in challenging potash strata. Furthermore, the presented approach and the conducted studies serve as an example for considering the application of unloading excavations in other geotechnical projects.

## imit state analysis of mining excavations

### FEM simulation setup

In this section, we describe a numerical simulation algorithm for a classic problem of the stress-stain state computation for a single mining excavation located in salt rock mass. Note that numerical solutions for this kind of problems are widely discussed in scientific literature^2,6,15,24,26,32–35^.

In this paper we use FEM to compute the stress-strain state of the considered geotechnical systems. We acknowledge that continuous methods like FEM cannot fully and directly simulate the behavior of such complicated inhomogeneous anisotropic media as rock mass and therefore the discontinuum methods such as DEM (Distinct Element Method) must be used to simulate the rock mass failure/fracture directly^36^. Such algorithms are described, for example in works^37,38^, one of the central publications of authors related to this research^34^, and references therein. However, the main aim of this study is not to simulate the fracture of the rock mass directly, but to investigate the transition of the rock mass to the limit state and its relief due to the use of unloading excavations. For this purpose, FEM simulations provide a consistent and sufficient approach.

The FEM simulation algorithm, which is used to compute the stress-strain state of the rock mass near the mining excavations, includes two main steps^33,34^:

1. **Compute the natural stress state of the unmined rock mass**. At this step the initial stress state of the rock mass is determined in static elastic formulation using the Hooke’s law. The computed stress state is then used as initial stress field for the step 2.
2. **Compute the stress-strain state of the rock mass after the excavation is created**. At this step the stress-strain state near the excavation is computed in elastoplastic formulation using the Hooke’s law and Coulomb-Mohr model.

**Fig. 1:**
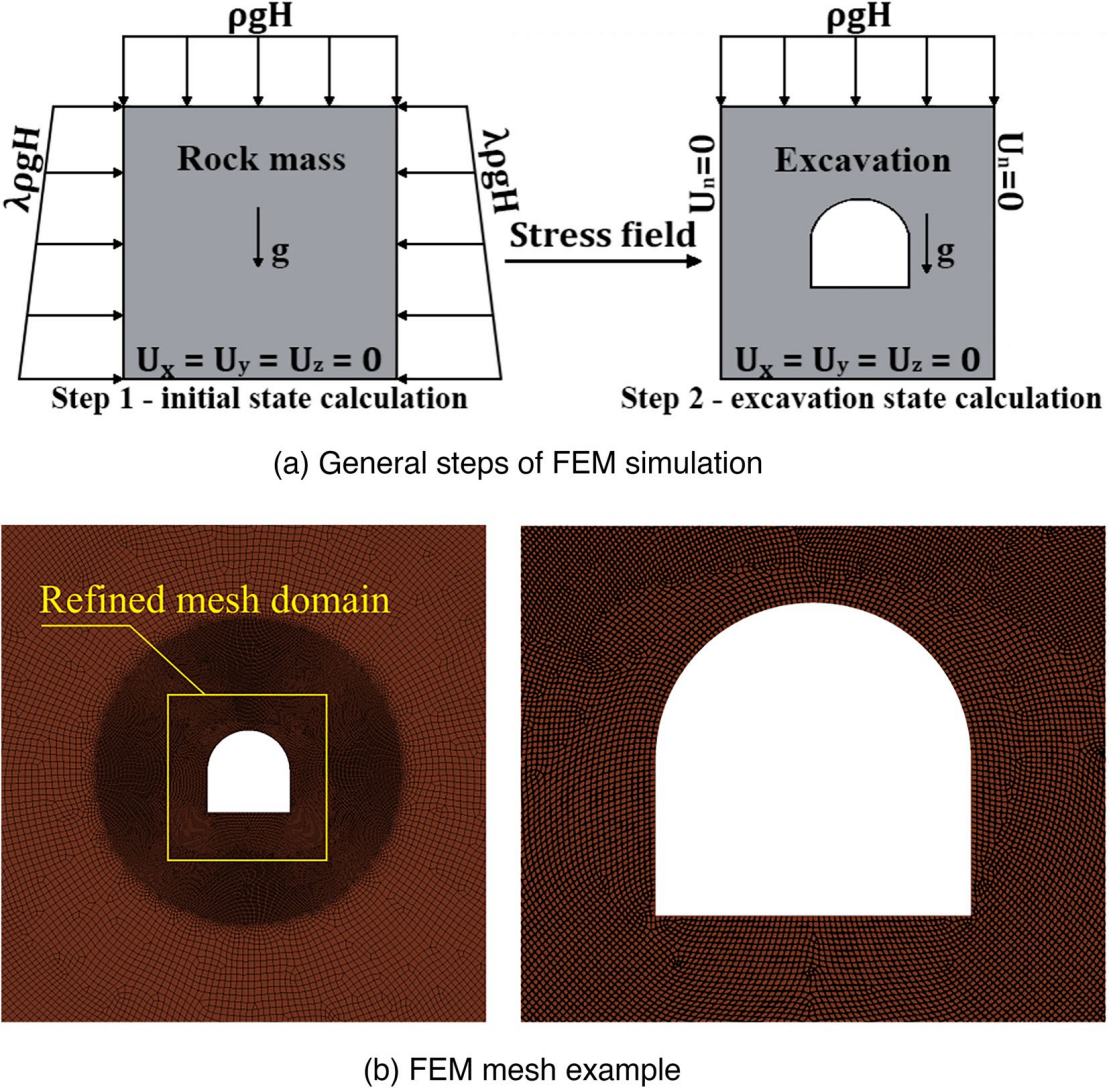
FEM simulation details for a single excavation.

These two steps, together with the corresponding boundary conditions, are schematically displayed in Fig. 1a below.

All the numerical experiments presented in this article are conducted according to this general algorithm with certain modifications for the case where unloading excavations are present, as described in Sect. 3. The model boundaries are removed at least 30 m from the study domain in each direction to simulate quasi-infinite boundaries of the rock mass. Therefore, the total area of the simulated rock mass is at least 60 by 60 m. Mostly 8-node quadrilateral elements are used for all simulations as displayed in (Fig. 1b). The element size varies from 5 cm in the study domain to 50 cm at the boundaries of the model. The total volume of the FE mesh is approximately 155 000 nodes in the cases of single excavations depending on its dimensions. The boundary conditions at the first step include the lithostatic pressure on the top of the model, the lateral pressure varied by depths, and displacement limitations at the bottom of the model. In addition to this, the self-weight of the model is taken into account. At the second step the natural stress field is used as initial and the lateral pressure is changed to normal displacement. Note that in (Fig. 1a) ρ is averaged density of the rock mass, *g* is gravitational constant, *H* is depth, *Un* is normal displacement and λ is lateral pressure coefficient^34^.

The combined elastic-plastic constitutive model used at the second step of the algorithm is a standard and computationally efficient approach for the limit state analysis in rock mechanics^1,2,33–35^. While it is recognized that salt rock exhibits a time-dependent creep, which is not captured by this constitutive relation, its use here is justified by a specific purpose of a systematic, comparative screening of excavation geometries under identical, conservative assumptions aiming to obtain the first-order approximations of the solution across a wide range of simulation cases.

The FEM analysis employs 2D plane-strain models for computational efficiency. While this approach cannot capture 3D effects, it is sufficient for establishing the fundamental mechanical behavior of excavations and assessing limit states across a wide range of parameters as shown in the references above^2,6,15,24,26,32–35^. In addition, the investigated excavations at the Starobin deposit can reach kilometers in length, therefore the 2D simplification is considered a necessary and acknowledged simplification that allows a systematic comparative study.

### Limit state calculation method

The limit state of a rock mass or geotechnical structure, refers to a condition in which the rock mass or structural elements experience significant violations of the limit state criteria, which can potentially lead to the formation of discontinuity zones, fracture, or crack propagation, without direct description of the critical phenomena^34^. Thus, the limit state does not directly imply rock mass failure or collapse but can serve as a representative comparative metric describing its proximity to failure. To assess the limit state of the rock mass, we use a comprehensive complex limit state criterion which was initially proposed in work^33^ and further developed, expanded and applied in work^34^. In this section we briefly discuss the definition and main features of this criterion. Specifically, the complex limit state criterion is not a new failure criterion, but it is a multicriterial framework which is designed to assess the limit state of the rock masses in complex stress-strain states based on existing verified and validated criteria. Thus, the complex criterion consists of a set of generally accepted geomechanical criteria, described, for instance, in works^39–41^. The main aim of this criterion is to provide a more accurate and physically realistic assessment of the rock mass’s proximity to failure than single-mechanism criteria. Its fundamental principle is that rock mass failure is not governed by a single mechanism but can occur through a combination of tensile fracture, shear slippage, or compressive crushing, depending on the local stress state type. Using a single failure criterion (e.g., Mohr-Coulomb or Drucker-Prager) for all stress states can lead to significant overestimation or underestimation of critical zones, as these criteria do not account for the fundamental differences in material behavior under different loading conditions^33^. In addition, even proven and established criteria can give very different predictions of limit state^33^.

We emphasize that the main advantage of the complex limit state criterion lies not in a fundamentally new approach to describing salt rock mechanics, but in its ability to combine the advantages of established criteria while limiting their disadvantages through their simultaneous use under a defined rule. The novelty of the complex limit state criterion lies in its structured, multi-mechanism approach. It first classifies the stress state at every point in the rock mass using the Nadai-Lode coefficient µ^33,34,42^, which serves as a stress-strain state type indicator (1):

1 $$\:{\upmu\:}=\frac{{2({\upsigma\:}}_{2}-{{\upsigma\:}}_{3})}{{{\upsigma\:}}_{1}-{{\upsigma\:}}_{3}}-1$$

The values of the Nadai-Lode coefficient are always in the interval $\:\left[-\mathrm{1,1}\right]$. This follows from the fact that $\:{{\upsigma\:}}_{1}\ge\:{{\upsigma\:}}_{2}\ge\:{{\upsigma\:}}_{3}$, therefore the boundary values of $\:{\upmu\:}=+1$ and $\:{\upmu\:}=\:-1$ are reached when $\:{{\upsigma\:}}_{2}={{\upsigma\:}}_{1}$ and $\:{{\upsigma\:}}_{2}={{\upsigma\:}}_{3}$ correspondently. In mechanics of materials such boundary values correspond to axisymmetric compression and axisymmetric tension, while $\:{\upmu\:}=0$ corresponds to pure shear. However, in such complicated natural objects as rock masses the stress state is very complex and almost never correspond to axisymmetric compression/tension/pure shear. Therefore, the interval $\:\left[-\mathrm{1,1}\right]$ is divided into three sub-intervals $\:\left[-1,-0.5\right)\cup\:\left[-\mathrm{0.5,0.5}\right]\cup\:\left(\mathrm{0.5,1}\right]$ corresponding to generalized compression, generalized shear and generalized tension respectively. The boundaries at $\:{\upmu\:}=-0.5$ and $\:{\upmu\:}=0.5$ are established to delineate the stress states where compressive, shear, and tensile failure mechanisms respectively become dominant, ensuring the most physically appropriate criterion is applied locally within the rock mass^33,34^.

After the Nadai-Lode-based segmentation, established criteria are applied in the corresponding areas of the rock mass. Mathematically, the complex limit state criterion is written as (2)^33,34^. Note that this formulation uses well-known Mohr-Coulomb or Drucker-Prager criteria for the limit state assessment of the rock mass in the areas of generalized share, along with other classical criteria in the areas of generalized compression and generalized tension. In addition to this, formulation (2) uses not only stress-based criteria, but strain-based criteria as well, which is crucial for rock masses, since the limit state can occur not only due to exceeding stress limits, but also as a result of exceeding the limit strain especially at great depths where the initial stress in the rock mass is close to the ultimate stress limit.

2 $$\:\left\{\begin{array}{c}\begin{array}{c}{[{\upsigma\:}}_{3}\le\:\:{{\upsigma\:}}_{\mathrm{c}},\:{{\upepsilon\:}}_{3}\le\:{{\upepsilon\:}}_{\mathrm{c}}]\:when\:\mu\:\in\:(0.5;1]\\\:\left[\left|{{\upsigma\:}}_{1}-{\uplambda\:}{{\upsigma\:}}_{3}\right|\le\:\:{{\upsigma\:}}_{c},\:\sqrt{{I}_{2}}\le\:{\upalpha\:}{I}_{1}+s,\:{{\upepsilon\:}}_{1}-{{\upepsilon\:}}_{3}\le\:{{\upepsilon\:}}_{s}\right]\:when\:\mu\:\in\:\left[-\mathrm{0.5,0.5}\right]\\\:{[{\upsigma\:}}_{1}\le\:\:{{\upsigma\:}}_{t},\:{{\upepsilon\:}}_{1}\le\:{{\upepsilon\:}}_{t}]\:when\:\mu\:\in\:(-0.5;-1]\end{array}\end{array}\right.$$

Here $\:{{\upsigma\:}}_{1}\:\mathrm{a}\mathrm{n}\mathrm{d}\:{{\upsigma\:}}_{3}$ are the maximum and minimum principal stresses; $\:{{\upsigma\:}}_{c},{{\upsigma\:}}_{t}$ are the ultimate compressive and tensile strengths; $\:{{\upepsilon\:}}_{1}$ and $\:{{\upepsilon\:}}_{3}$ are the maximum and minimum principal strains; $\:{{\upepsilon\:}}_{\mathrm{c}}$, $\:{{\upepsilon\:}}_{t}$, $\:{{\upepsilon\:}}_{\mathrm{s}}$ are the ultimate compressive, tensile and shear strains; $\:{{I}_{1},I}_{2}$ are the first and the second invariants of the stress tensor, $\:{\upphi\:}$ is inner friction angle, *С* is cohesion; $\:{\upalpha\:}=\frac{2\mathrm{sin}{\upphi\:}}{\sqrt{3}(3-\mathrm{sin}{\upphi\:})}$; $\:s=\frac{6\:C\mathrm{cos}{\upphi\:}}{\sqrt{3}(3-\mathrm{sin}{\upphi\:})}$, $\:{\uplambda\:}=\frac{1+\mathrm{sin}{\upphi\:}}{1-\mathrm{sin}{\upphi\:}}=\frac{{{\upsigma\:}}_{c}}{{{\upsigma\:}}_{t}}$, $\:\mu\:$ is the Nadai-Lode coefficient^33,34^.

Thus, in practical applications described below, the limit state assessment follows a systematic algorithm^33,34,43^:

1. Calculate stress state using FEM as described in Sect. “FEM simulation setup”.
2. Compute $\:{\upmu\:}$ at each point using (1).
3. Apply the appropriate sub-criterion from (2) based on $\:{\upmu\:}$ value.
4. Aggregate results to identify the resulting limit state zones as unification of zones obtained by all criteria from (2).

We emphasize that the described complex limit state criterion is not a new constitutive relation for salt rock masses, but a powerful tool for the synergetic limit state analysis based on the stress-strain state obtained as a result of FEM simulations as described in Sect. “FEM simulation setup”. In this regard, the results below do not provide the exact distributions of the particular stress or strain tensor components as well as the values of the safety factors according to each particular criterion. Instead, the results show the distributions of the limit state zones according to the described algorithm, allowing comprehensive aggregated comparison of the rock mass state near the underground structures.

### Mechanical properties of the rock mass

The mechanical properties of rock masses used for numerical simulations in this paper are presented in the Table 1 below^34^. The values in Table 1 are obtained from externally performed laboratory tests on rock specimens taken from the Starobin potash deposit^34^. The presented parameters are standard and defined according to an established salt rock specimen testing methodology, described, for example, in work^44^. Therefore, in this paper we do not provide a detailed description of specimen preparation and testing, since this study focuses on establishing the essential principles and methods for the design of unloading excavations. However, another geological conditions and rock mass properties may require recalibration of the model, but the core framework we describe is widely applicable.

**Table 1:** Mechanical properties of the rock masses used for numerical simulations.

	Rock salt 300 m	Rock salt 600 m	Rock salt 900 m	Rock salt 1200 m	Clay	Sylvinite
Density, kg/m^3^	2300	2300	2300	2300	2150	2300
Ultimate compressive strength, MPa	21	25	28	35	7	32
Ultimate tensile strength, MPa	1	1	1,2	2	0.5	1
Ultimate compressive strain, %	2	2	2	2	2	2
Ultimate tensile strain, %	0.4	0.4	0.4	0.4	0.4	0.4
Young’s modulus, GPa	1.65	1.75	1.9	2	0.56	1.64
Poisson’s ratio	0.25	0.28	0.29	0.35	0.4	0.29
Inner friction angle,degree	40	48	53	60	30	70
Cohesion, MPa	2.4	3.8	4.2	5	1.25	2.83

### Results of limit state analysis for single mining excavations

**Fig. 2:**
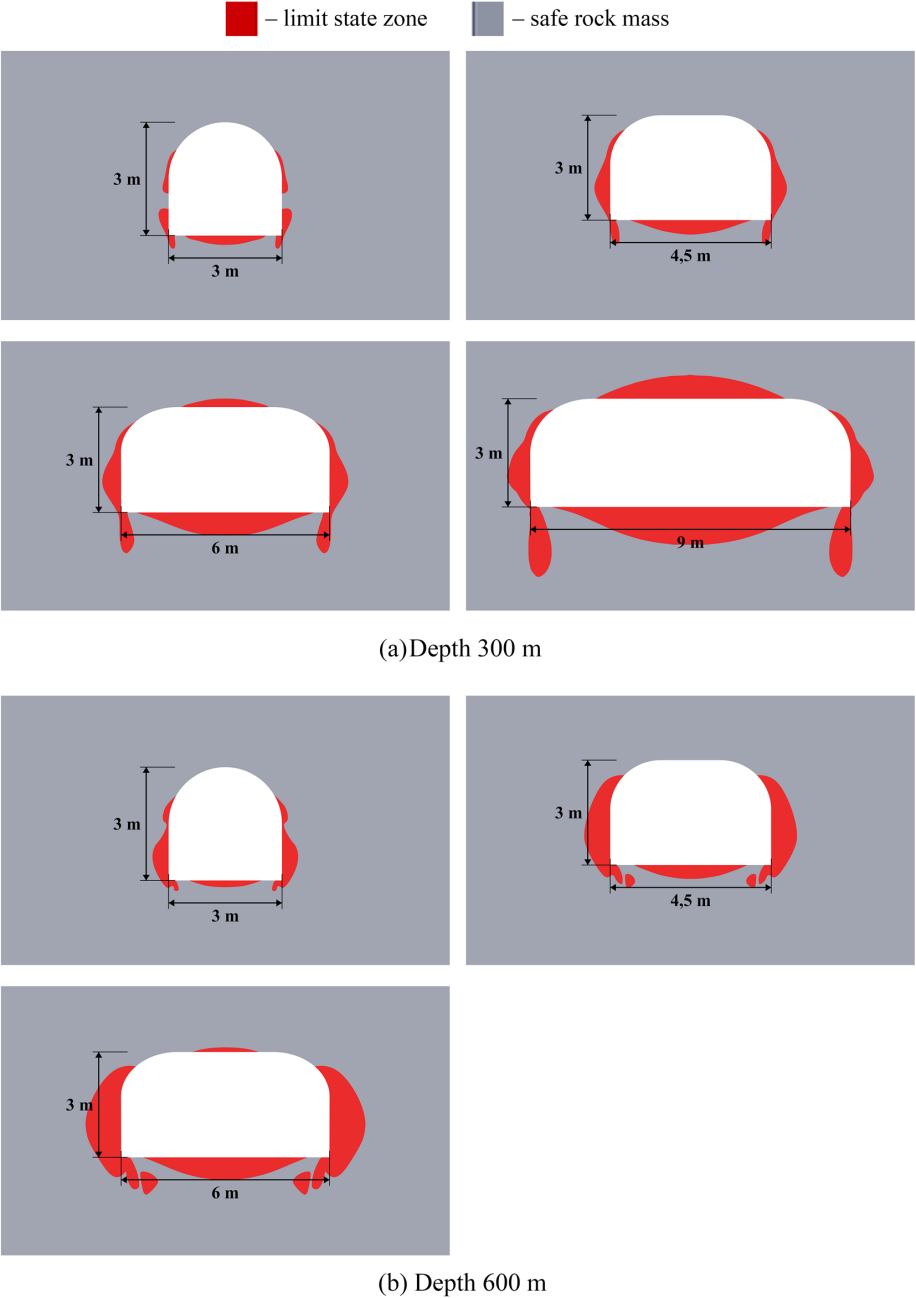

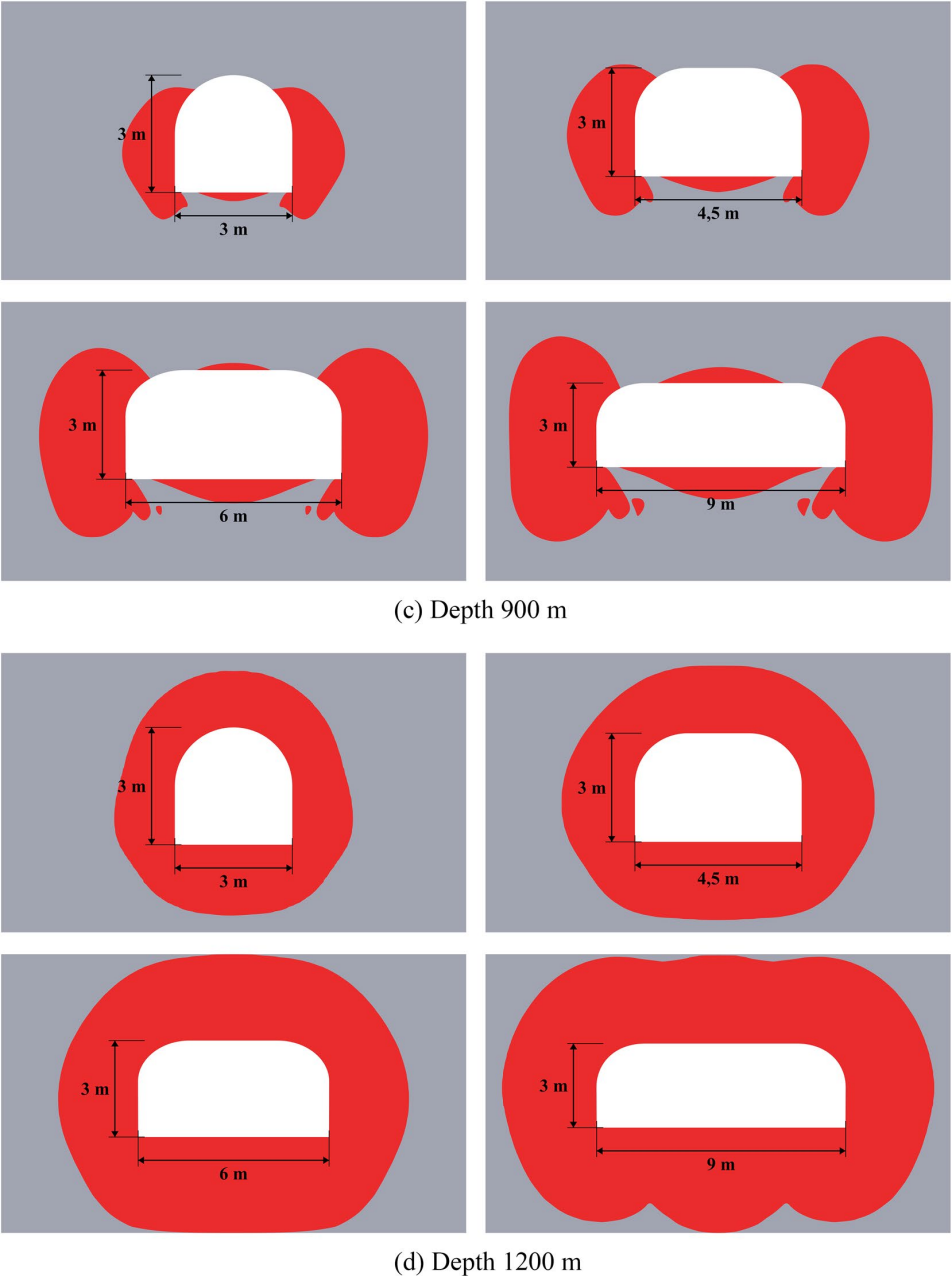
Limit state zones in the vicinity of single mining excavations at different depths.

Results of limit state analysis for single mining excavations located at different depths are presented below in Fig. 2. This figure demonstrates that for small (300 m) and great (1200 m) depths the limit state zones significantly differ. Thus, at great depths, closed limit state rings form in the vicinity of excavations, which may be caused by the zonal disintegration effect discovered in the late 20th century^45,46^. This effect involves the formation of alternating concentric rings of fractured and intact rock around excavations when the gravitational stress approaches or exceeds the rock’s compressive strength. Such zonal failure is a fundamental natural process that allows cavities to exist at great depths. Here we acknowledge the importance of this effect; however, the study of zonal disintegration is not the aim of this article. The limit state zones at small and moderate (600–900 m) depths differ more quantitatively than qualitatively. The depths less than 300 m are not considered in this paper as they are irrelevant for Starobin potash deposit. More specific information about depth classification into small, moderate, and great can be found in work^34^.

The dimensions of the limit state zones are summarized in Table 2. The linear extent of these zones, reported in meters, is defined as the perpendicular distance from the excavation boundary to the limit state frontier. This metric is prioritized as it provides a direct input for designing targeted safety measures, such as determining the required length of rock bolts at specific locations along the excavation. For comprehensive analysis, the total area of the limit state zone is also provided as a standard metric.

**Fig. 3:**
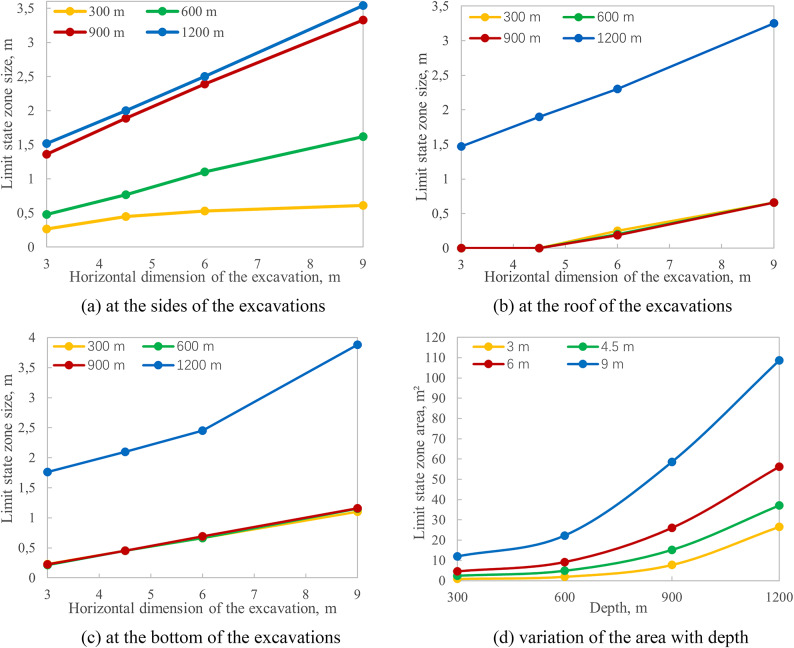
Comparison of the limit state zone size near single mining excavations.

The data of Fig. 2 and Table 2 show that the size of limit state zones nonlinearly grows with the increase of mining depths and horizontal dimension of the excavation. This fact is graphically illustrated in Fig. 3. When transitioning from moderate to great depths, the limit state zone size becomes approximately the same at the roof, sides, and bottom of the excavations, forming a connected limit state region around the excavation outline. The topology of limit state zones significantly changes as shown in Fig. 2.

**Table 2:** Comparison of the limit state zones for single excavations.

Horizontal dimension of the excavation, m	Depth, m	Roof limit state size, m	Bottom limit state size, m	Side limit state size, m	Total area of the limit state zone, m^2^
3	300	0	0.23	0.265	0.89
4.5		0	0.45	0.45	2.44
6		0.25	0.66	0.53	4.6
9		0,66	1.1	0.61	11.97
3	600	0	0.215	0.48	1.96
4.5		0	0.45	0.77	4.89
6		0.2	0.66	1.1	9.19
9		0.66	1.15	1.62	22.28
3	900	0	0.226	1.362	7.8
4.5		0	0.45	1.89	15.2
6		0.185	0.69	2.392	26.05
9		0.66	1.16	3.33	58.61
3	1200	1.47	1.76	1.52	26.62
4.5		1.9	2.1	2	37.13
6		2.3	2.45	2.5	56.22
9		3.25	3.88	3.54	108.73

This section presents the computed limit state zones, which depict regions where the rock mass meets the complex limit state criterion, rather than directly simulating rock fracture or collapse processes as discussed in Sect. “FEM simulation setup”. This approach is sufficient for comparing single excavation scenarios with the unloading excavation efficiency analysis in Sect. 3, as the extent and configuration of these zones correlate strongly with potential failure regions and provide a conservative estimate of their extent. For direct simulation of rock fracture and subsequent collapse in site-specific geotechnical assessments, we recommend employing the coupled FEM-DEM algorithm presented in^34^.

### Validation of the results for a single excavation

Validation of the presented results is a nontrivial task due to limited field data and the difficulty of conducting experiments in situ, especially at great depths over 900 m. The solution algorithm, described in Sect. “FEM simulation setup” and “Limit state calculation method”, has been verified in previous publications^47,48^ against excavation stress-strain states during nearby longwall mining operations and against corresponding Earth surface subsidence. To validate the results of this particular study presented above, further we compare the displacements of the excavations outline obtained from numerical simulations with in-situ measurements at the mining excavations of Starobin potash deposit. To obtain this data, various mining excavations at depths of 870–920 m in the mentioned deposit were monitored via periodic measurement of roof, sidewall and bottom rock mass deformation. Measurements at small and moderate depths (300–600 m), while feasible, were not a priority for this study as the limit state at these depths differs quantitatively rather than qualitatively from 900 m validation case presented below. Furthermore, data for great depth of 1200 m is unavailable due to the absence of stable excavations at such depth at the Starobin deposit. Consequently, investigating the feasibility of creating stable excavations at great depths is one of the key objectives of the present research, aimed to be achieved with the help of unloading excavations.

As is well-known, salt rock masses tend to demonstrate rheological behavior, resulting in creep deformation and stress relaxation. Therefore, to compute the long-term deformation of the excavation outline we employ a two-staged creep model (3)^49^. The rheological constants for this model are given in Table 3 below. These creep constants were obtained using the curve fitting method and the data of rock salt specimen time-dependent deformation^49^.

3 $$\:{\upepsilon\:}\left(t\right)=\frac{1}{{C}_{3}+1}{C}_{1}{{{\upsigma\:}}_{eqv}}^{{C}_{2}}{t}^{{(C}_{3}+1)}+{C}_{4}{{{\upsigma\:}}_{eqv}}^{{C}_{5}}t,$$

where $\:{C}_{1}-{C}_{5}$ are creep constants, $\:{{\upsigma\:}}_{eqv}$ is equivalent stress, *t* is time.

**Table 3:** Creep constants of the salt rock mass for the model (3)^48^.

$\:{\boldsymbol{C}}_{1}$	$\:{\boldsymbol{C}}_{2}$	$\:{\boldsymbol{C}}_{3}$	$\:{\boldsymbol{C}}_{4}$	$\:{\boldsymbol{C}}_{5}$
2*10–35	4.2	-0.7	5.1*10–38	0.2

**Fig. 4:**
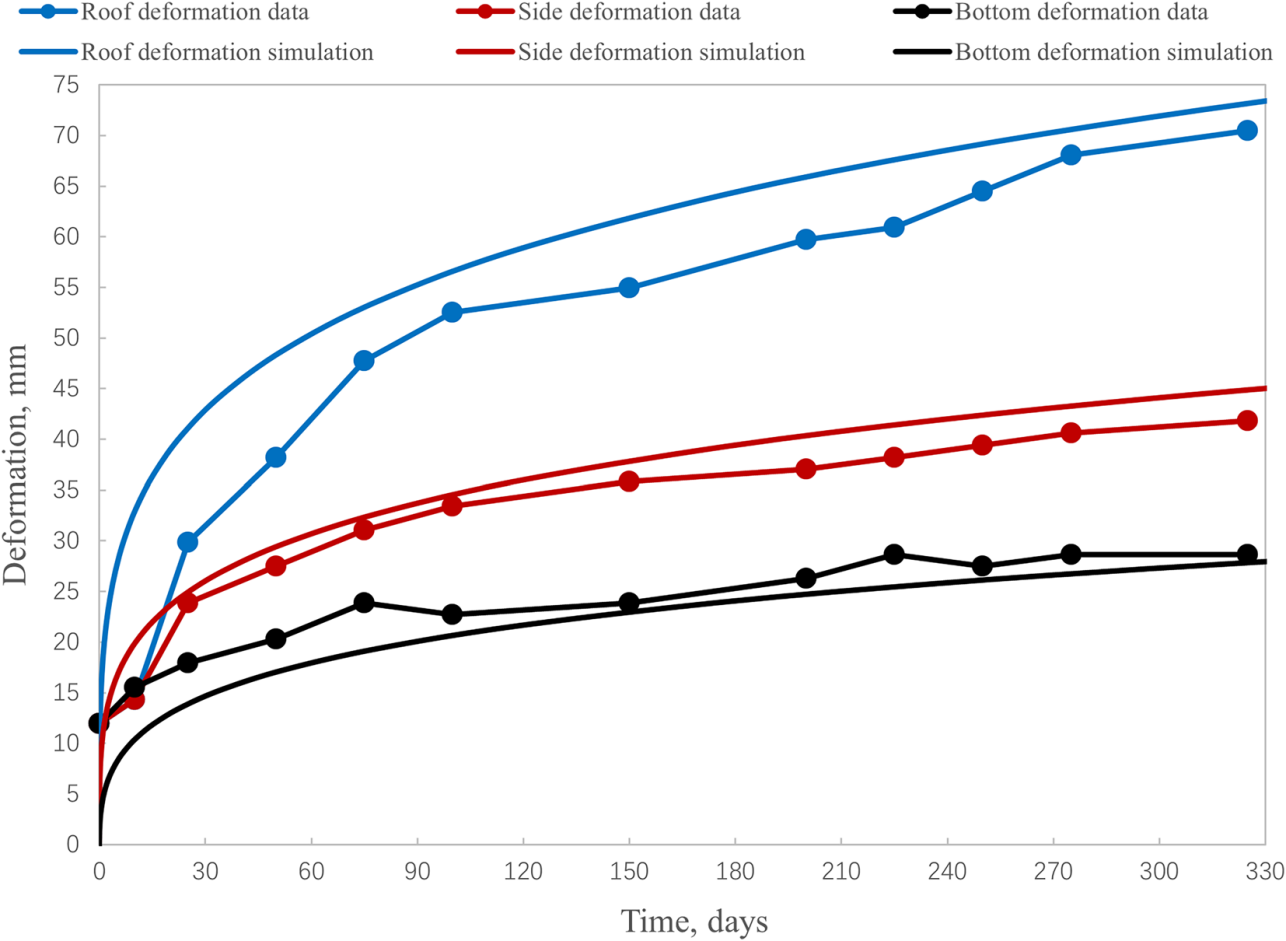
Validation of the simulation results against the deformation field data for a 3 m excavation obtained for the depth of 900 m.

To validate the results of the stress-strain state computations, we incorporated the creep model (3) into the FEM simulation to obtain the full time-dependent deformation of the excavation outline over the observed period. A comparison of the simulated long-term excavation outline deformation with field data is presented in Fig. 4. The simulation results of Fig. 4 are obtained by adding another step to the algorithm, described in Sect. “FEM simulation setup”. This step employs the near-excavation stress-strain state obtained during static simulation and uses creep model (3) to compute long-term stress-strain field.

Figure 4 shows that in general the numerical simulation results fit the field data well at least after 1 year after the excavation is created. The average relative computation error is approximately 13% for the roof deformation, 9% for the side deformation, and 12% for the bottom deformation. The most noticeable discrepancy between the simulation and field data is observed during the initial 70-day period of primary creep, which is characterized by high strain rates^49,50^. During this transient phase, the peak relative error reaches 56% for roof deformation, 28% for the side deformation, and 32% for the bottom deformation within the first 10 days. However, these errors rapidly diminish within the 70 days, resulting in an average model accuracy of 87–91% over a one-year period.

While the results in Fig. 4 do not directly validate the extent of the limit state zones shown in Fig. 2 and the subsequent results in Sect. 3, they do demonstrate that the numerical model accurately captures the displacement field of a real underground excavation in a salt rock mass. The model achieves good accuracy during the first year after excavation, providing robust validation for deep geotechnical structures under complex conditions. This high level of agreement establishes confidence in the reliability of the computed stress-strain state, which forms the basis for the limit state assessment.

We acknowledge that this study decouples the time-independent limit state analysis from the time-dependent creep behavior of the salt rock. This approach is justified as our primary goal is to assess the short-term limit state (typically 6–18 months in potash mining^50^, for which the model is validated. Consequently, the limit state zones should be interpreted as a conservative, short-term indicator of potential failure initiation under a worst-case scenario, as they do not account for long-term stress relaxation.

Furthermore, Fig. 4 indicates that deformations stabilize approximately 25–70 days after excavation. Mechanically, this stabilization corresponds to the salt rock’s transition from primary to secondary creep, characterized by a significantly reduced strain rate. Therefore, mining operations, including the creation of the unloading excavations described in the next section, should be scheduled after this stabilization period.

## Analysis of the unloading excavations effectiveness

### Unloading excavations in homogeneous rock mass

**Fig. 5:**
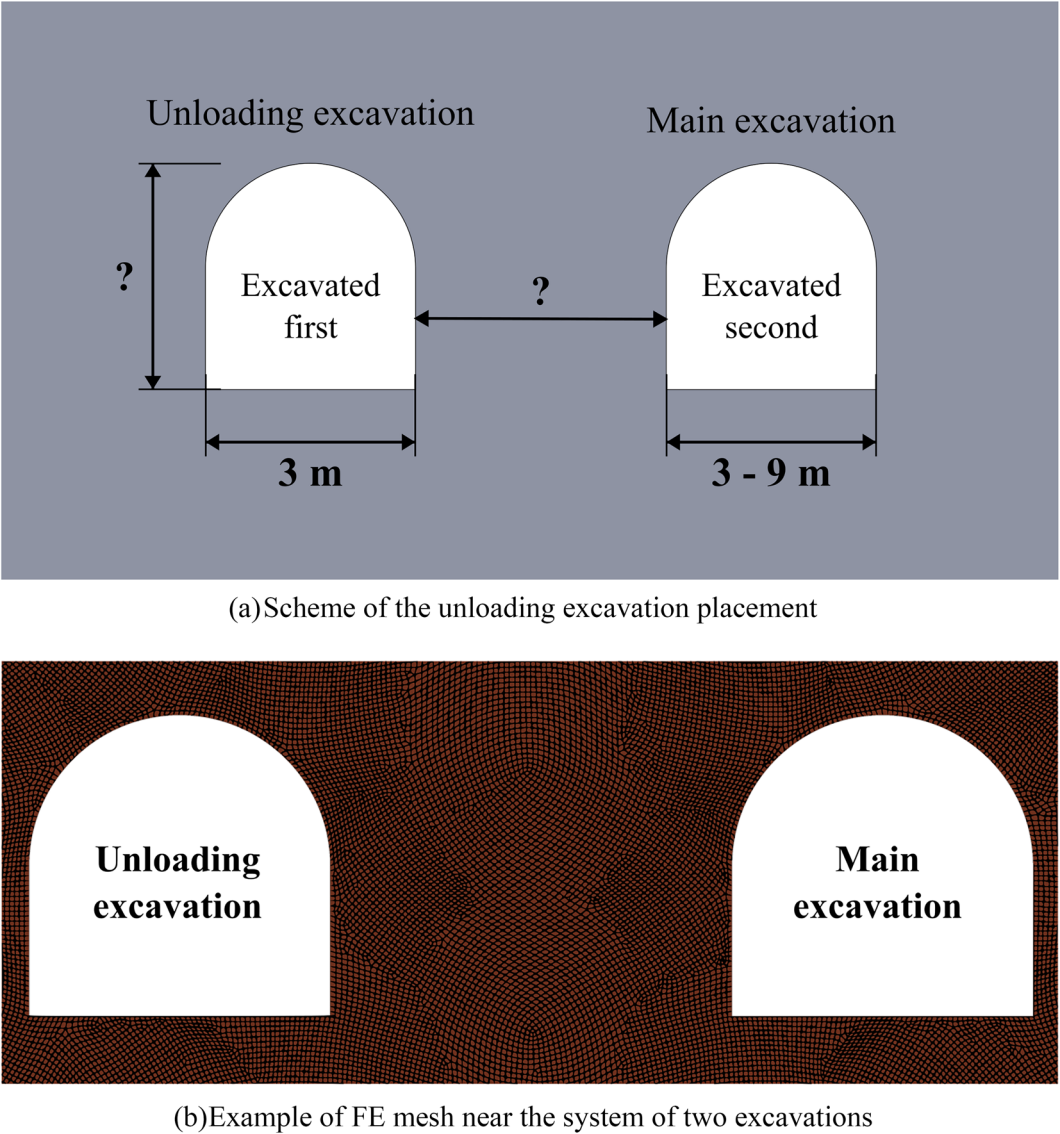
Scheme of the unloading excavation placement and meshing.

In this section, we investigate the effectiveness of unloading excavations by means of limit state analysis in various scenarios, focusing primarily on the spacing between excavations. The general scheme of the considered geotechnical systems is shown in (Fig. 5a). Note that in Fig. 5, the unloading excavation is created before the main excavation. According to the outcomes of Sect. “Validation of the results for a single excavation”, the time period between the creation of the unloading and main excavations should be no less than 25–70 days, to avoid the primary creep of the salt rock. However, in this paper we do not simulate the time-dependent behavior of unloading excavations directly, therefore the further simulations are conducted in quasi-static statement. Thus, it is considered that before the creation of the main excavation the outline of the unloading excavation passed the primary creep stage and is in the equilibrium state. The distance between excavations, the horizontal dimension of the main excavation, and the heights of the unloading excavation varies as shown in (Fig. 5a). Establishment of the optimal time lag between excavations requires separate study.

To simulate the behavior of the system of excavations in Fig. 5, the algorithm from Sect. “FEM simulation setup” is used with one crucial change: the second general step is subdivided into two substeps. At the first sub-step the stress-strain state induced by the unloading excavation is calculated. At the second substep the main excavation is created and the resulting stress-strain state is calculated. This is achieved by switching off the corresponding elements. The volume of the FE mesh in this group of numerical experiments is approximately 175 000 nodes depending on a particular case study. The FE mesh in the study domain is shown in (Fig. 5b).

**Fig. 6:**
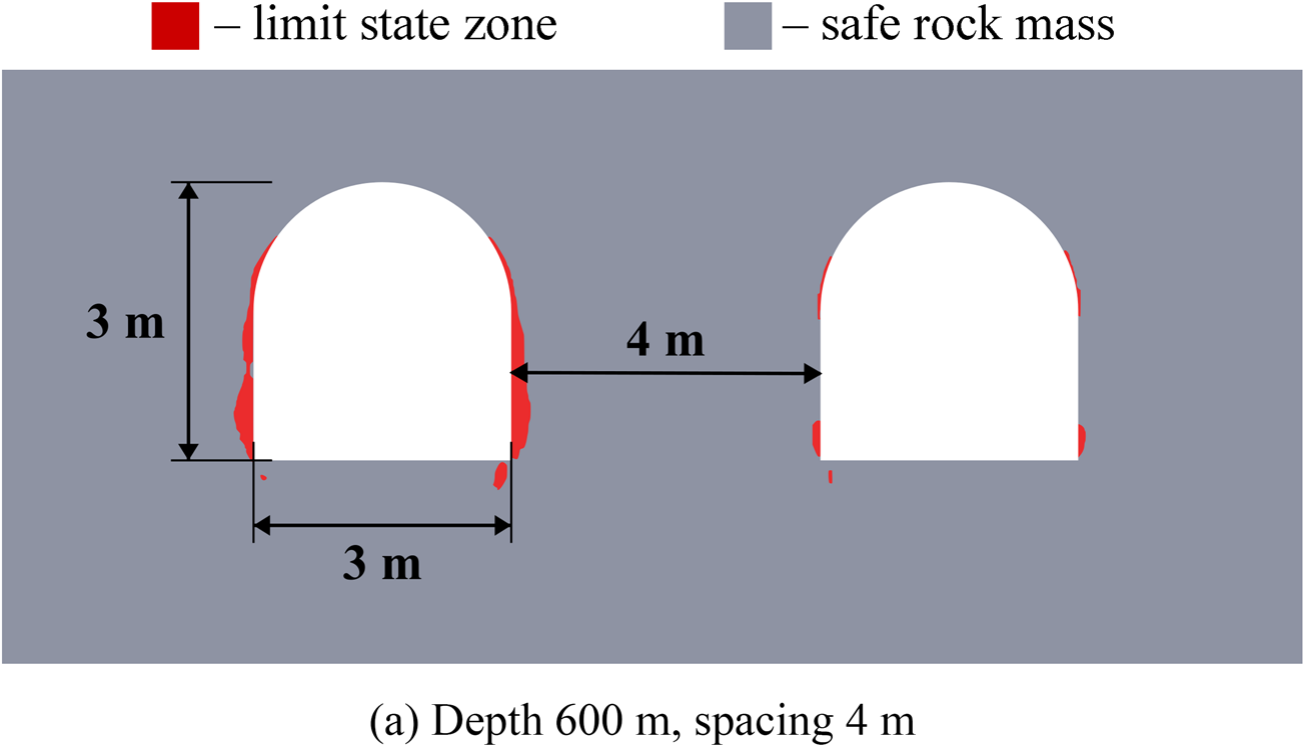

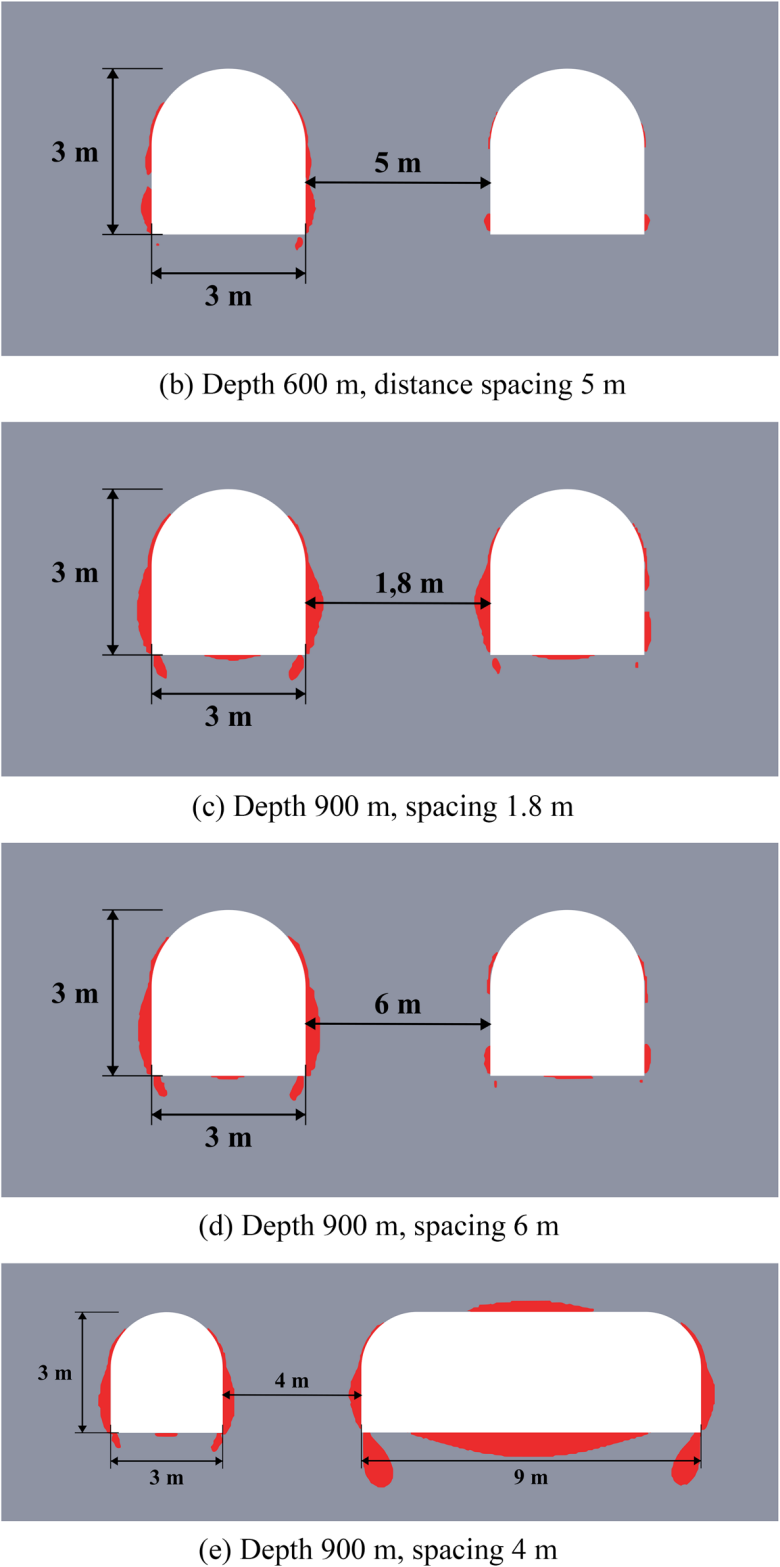

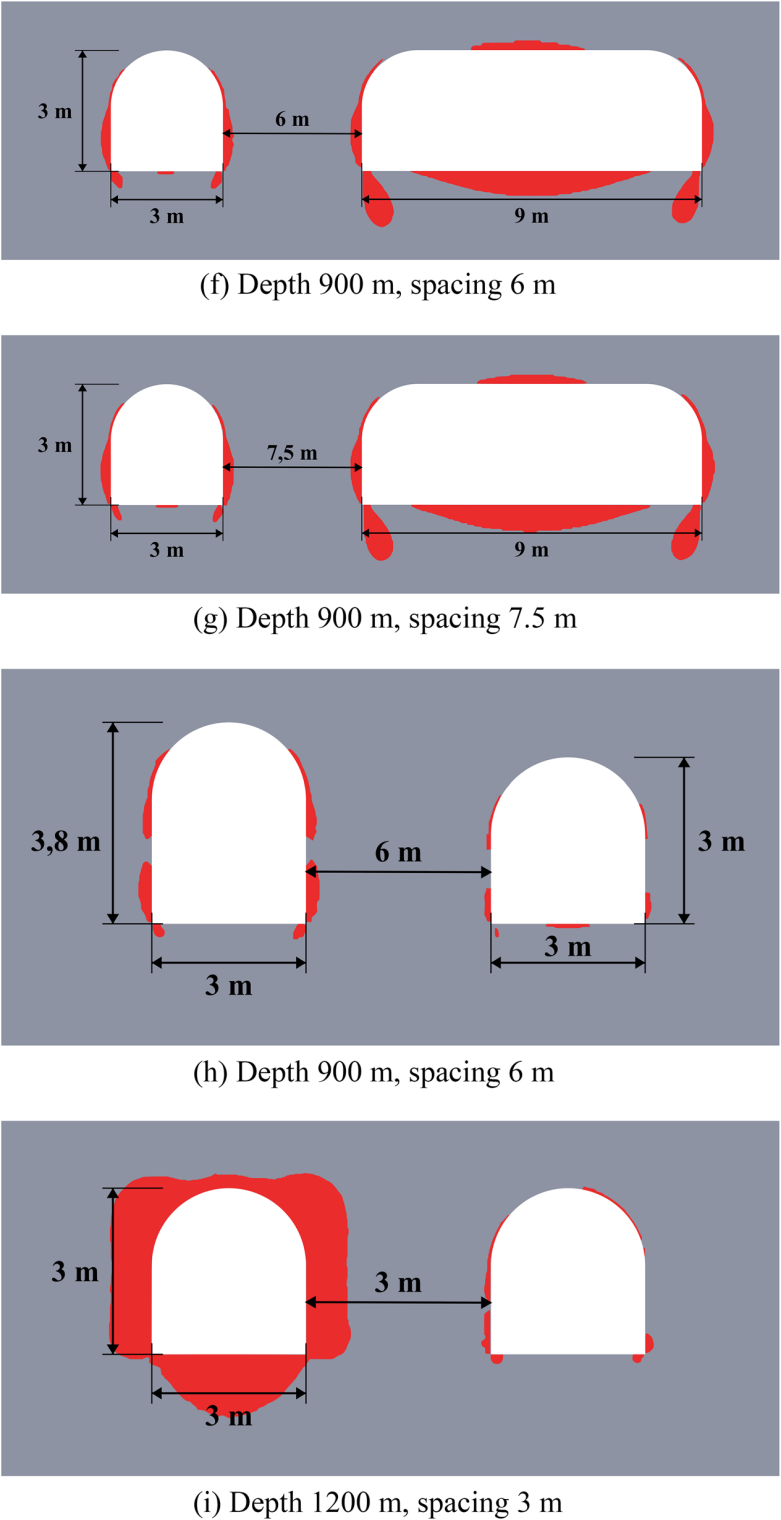

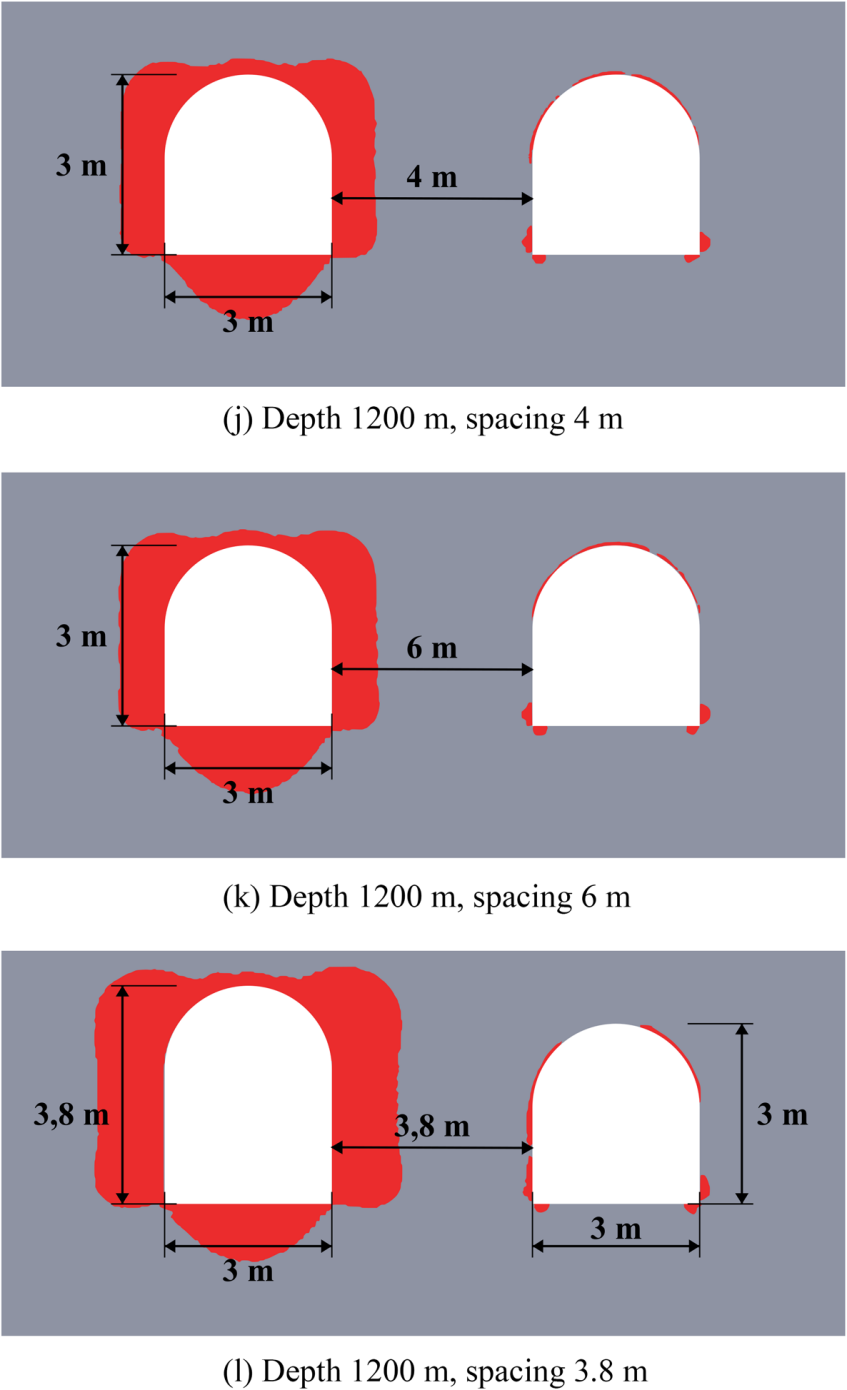

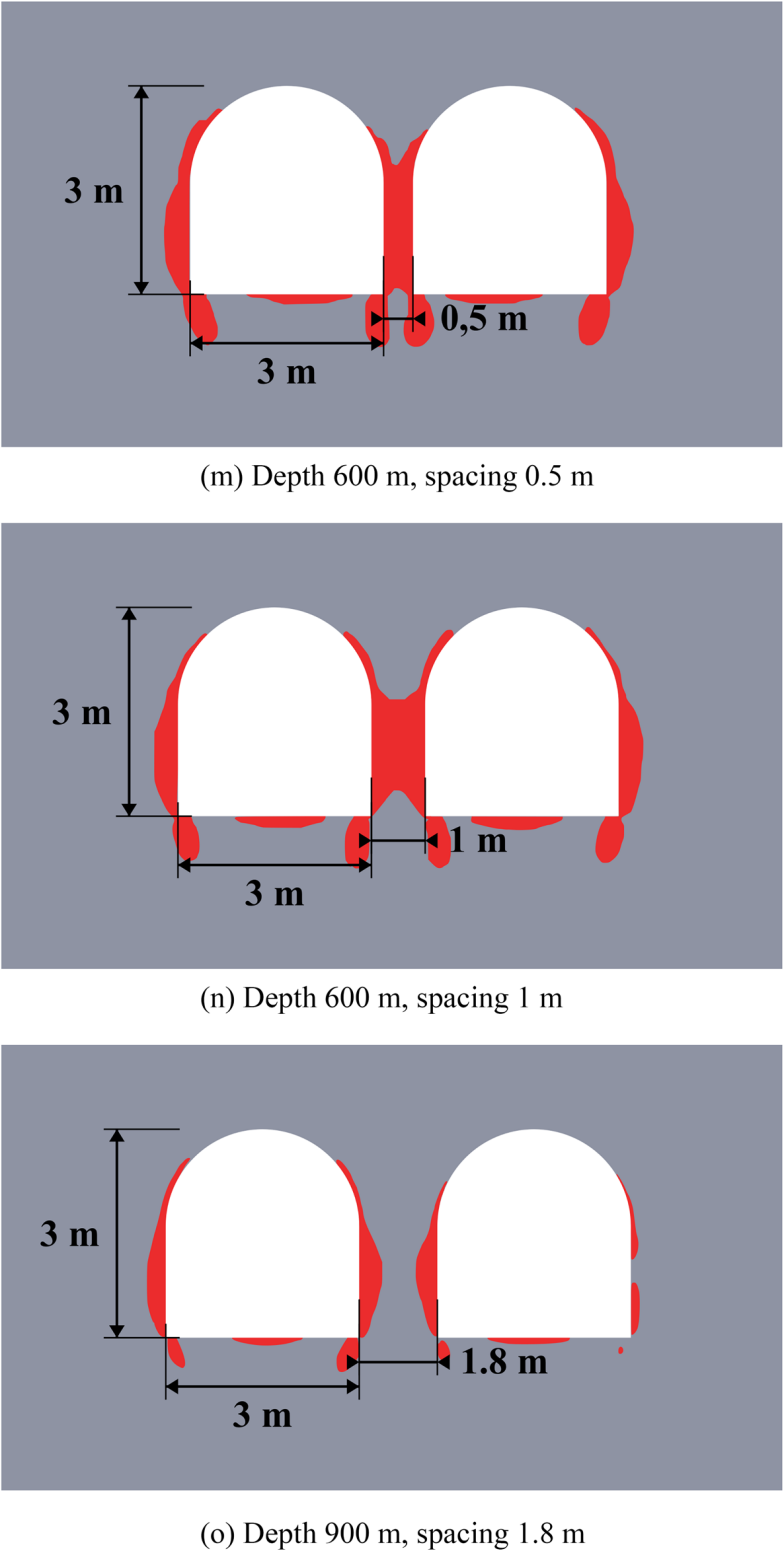

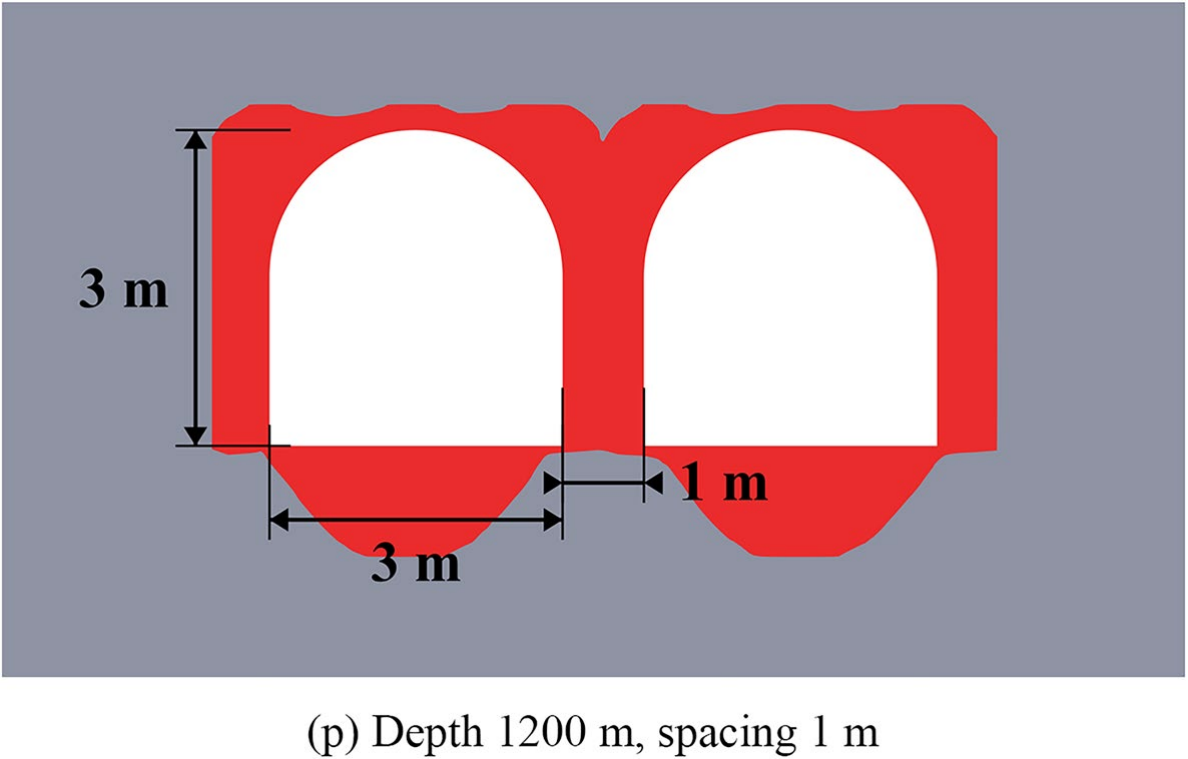
Limit state zones in the vicinity of geotechnical system of unloading and main excavations.

Figure 6 below depicts the limit state zones around a system of two excavations, where the key variable is the distance between a constant 3-meter-wide unloading excavation (left) and a variably-sized main excavation (right). This experimental setup aims to identify the most efficient spacing within a relatively homogeneous rock mass. The unloading excavation’s horizontal dimension was held at this specific 3-meter minimum, as defined by mining technology constraints at the Starobin deposit. While a larger unloading excavation is technically possible, it would induce greater stress concentrations and expand its own limit state zone (see Fig. 2), ultimately forcing a larger spacing that would diminish the unloading effect on the main excavation and increase operational costs.

The data from Fig. 6 demonstrates that the strategic placement of unloading excavation can significantly reduce the limit state zones around a main underground structure. This results not from the direct decrease of the individual stress components, but from a significant redistribution of the entire stress-strain state and the consequent change in its type (generalized compression, shear or tension) at every point within the considered region of the rock mass. Effective unloading excavations placement is governed by a specific distance interval. If the unloading excavation is too close, specifically less than 3.5 m at moderate depths or less than 5.5 m at great depths (over 900 m), the limit state zones and stress concentrations from the two excavations will intersect. This interaction not only cancels the unloading benefit but can also compromise the stability of the entire system. On the other hand, if the distance exceeds 5.5 m at moderate depths or 7 m at great depths, the unloading effect progressively weakens and eventually vanishes. This defines a clear optimal interval for placing the unloading excavation to ensure its effectiveness in decreasing the limit state zones.

Another important finding from this group of numerical experiments is that unloading excavations primarily help to decrease shear stresses, while the reduction in tensile and compressive stresses is limited to only 8–10%. This conclusion is drawn from a separate analysis of the individual failure criteria in system (2). Furthermore, analysis of individual stress and strain components reveals significant discrepancies in how they define the extent of limit state zones, thereby demonstrating the necessity of a comprehensive assessment approach. This is particularly critical at significant depths where initial values of individual stress components may already exceed the rock’s strength limits, justifying the use of complex limit state criterion as a synergetic comparative tool for limit state analysis.

The efficiency of unloading excavations is summarized in Table 4. The data indicates that optimal placement of the unloading excavation leads to a measurable decrease in the extent of limit state zones (from 11% to 100%). At small and moderate depths the size of the limit state zones decreases up to 2 times. While the unloading effect diminishes significantly (by 1.9–8.8 times) with increasing depth, it remains present even at depths as great as 1200 m. Furthermore, the mechanism of unloading shifts with depth. At small moderate depths, limit state zone decrease occurs primarily in the roof of the excavations (86–100%). In contrast, at greater depths, the sides experience more unloading (40%) than the roof or floor (11–14%). This shift is likely attributable to the increasing dominance of lateral rock pressure over vertical stress at great depths.

These observations are particularly important for deep mining projects, as the efficiency of unloading excavations is highly depth-dependent. At small depths (less than 300 m), limit state zones are typically localized, and their mitigation can often be achieved using traditional, more cost-effective safety measures; therefore, the implementation of unloading excavations may not be economically justified. In contrast, at moderate and great depths, limit state zones become vast, potentially form closed loops, and extend far from the excavation boundary. This makes unloading excavations a highly effective and economically viable solution, as their benefits in controlling large-scale mechanisms outweigh the associated costs.

**Table 4:** Generalized unloading effect values for the homogeneous rock mass.

Depth (m)	Limit state zone decrease in the excavation side, %	Limit state zone decrease in the excavation roof, %	Limit state zone decrease in the excavation bottom, %	Decrease of the limit state zone total area, %
300	73.2	100	100	60.7
600	72.4	100	86.3	73.0
900	43.3	85.7	31.3	86.3
1200	39.5	14.1	11.4	65.2

### Unloading excavations in multilayered rock mass

In this section, we evaluate the effectiveness of unloading excavations in a multilayered rock mass, since in a wide range or real applied cases the rock mass is inhomogeneous and cannot be treated as homogeneous isotropic media. This issue makes the simulation significantly more complex due to scale effect and different mechanical properties of geological layers. Such conditions are also typical for Starobin potash deposit, where the capacity of thin sylvinite and clay layers can vary from 5 to 20 cm.

**Fig. 7:**
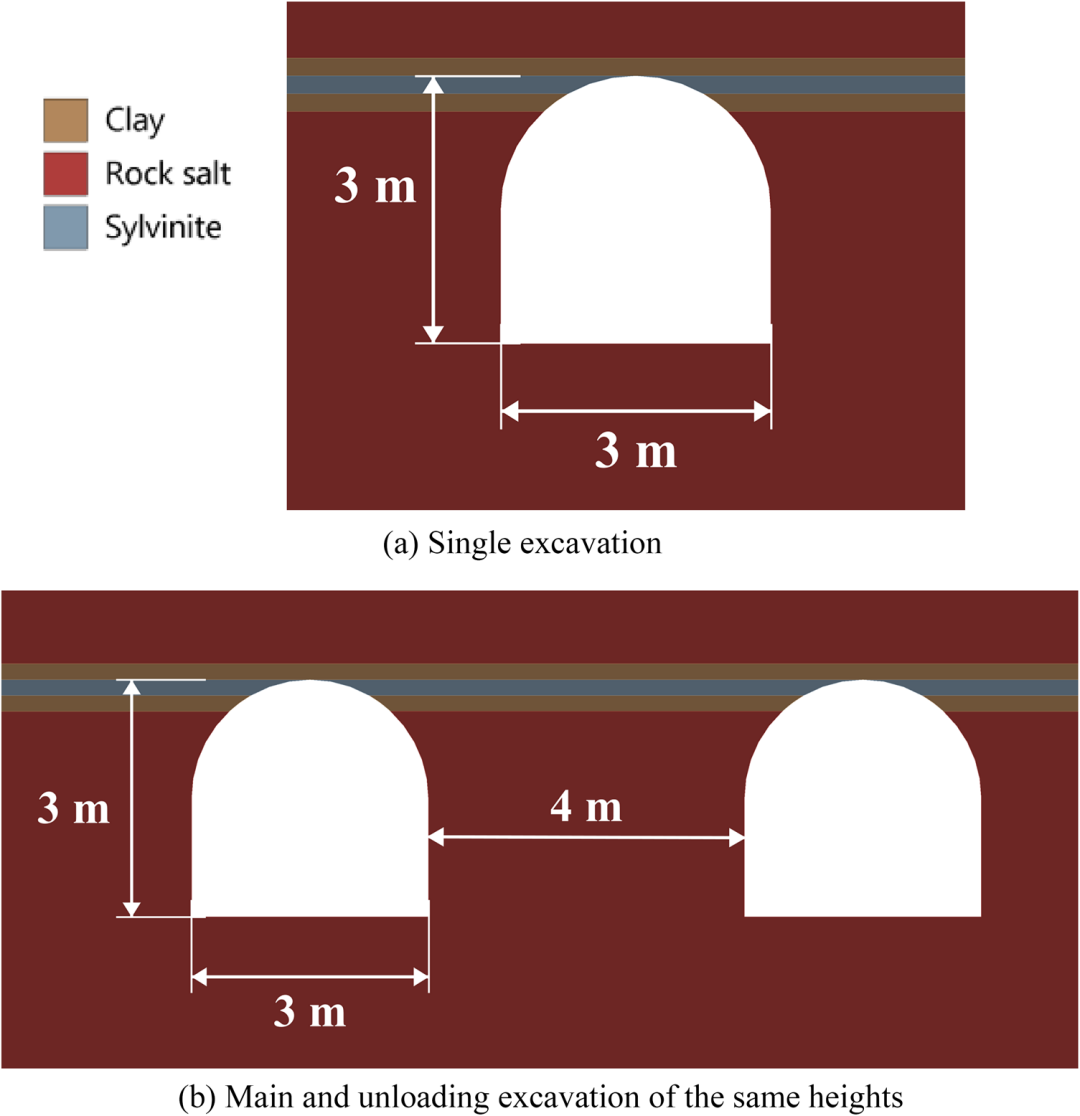

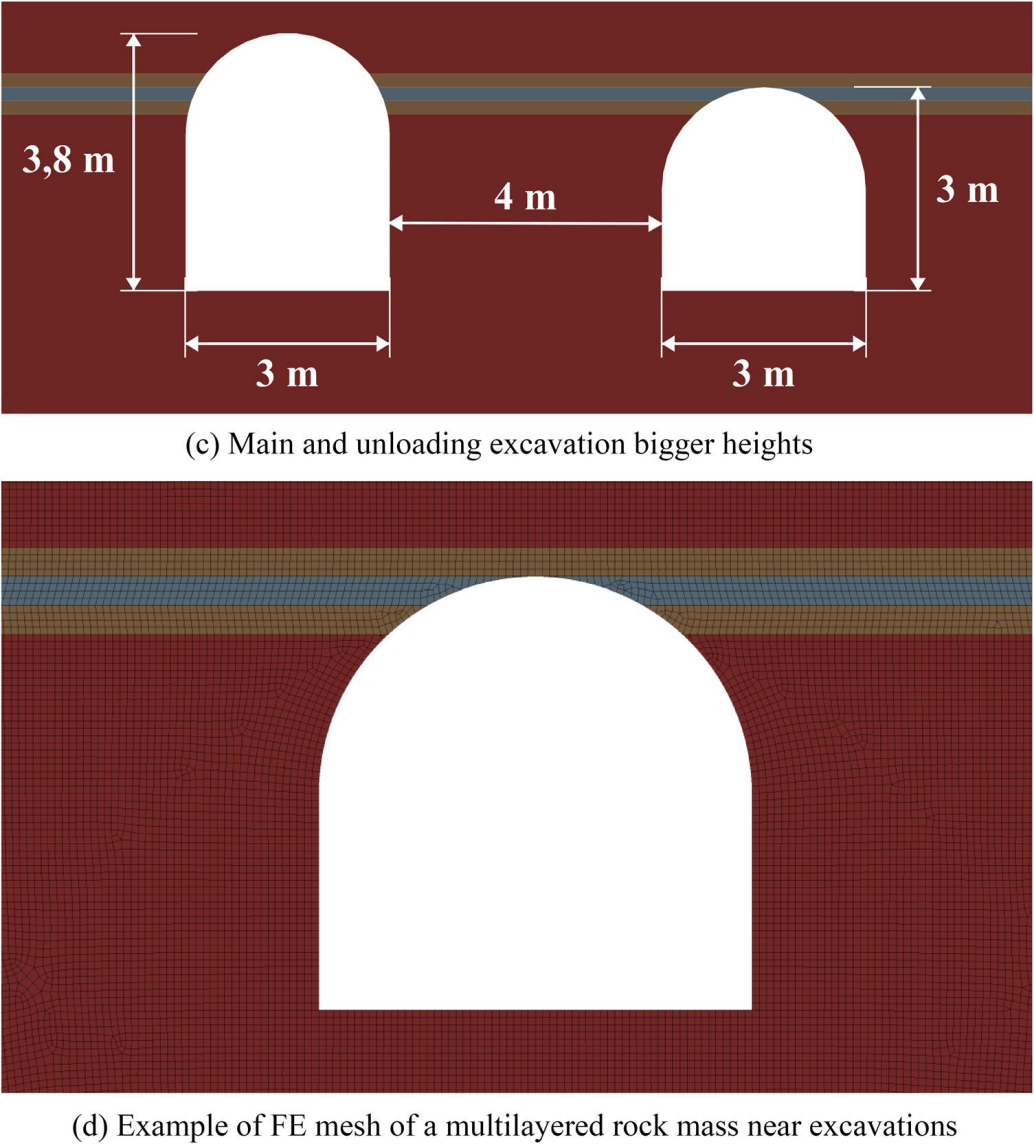
Scheme of the unloading excavation placement and meshing in multilayered rock mass.

The schemes of the considered cases are presented in Fig. 7 below. The geological structure of the rock mass includes five geological layers of various capacities. The excavations are located in rock salt, while there are some small layers of clay and sylvinite in the roof of excavations. This group of numerical experiments investigates the most effective unloading technique for the multilayered rock strata by varying the spacing between excavations and the height of the unloading excavation.

A key simplification in this model is the assumption of perfect bonding between geological layers, which excludes the potential for debonding or direct shift at the interfaces. While this approach is a simplification of the layered media behavior, since it may struggle to capture interface-dominated failure, the high level of mesh refinement effectively mitigates numerical inaccuracies for general stress-strain state computation. To enhance accuracy in these critical zones, the FE mesh was significantly refined to an element size of 3 cm within the geological layers, increasing the total number of nodes to approximately 200 000 as visualized in (Fig. 7d). We emphasize that the results of this section are not intended to provide fully accurate computations for specific cases with particular locations of geological layers and their properties. However, the considered configuration is deemed fully sufficient for the primary goal of this study, which is to determine the fundamental development of limit state zones during the unloading process, providing critical and reliable insights into the overall system response.

**Fig. 8:**
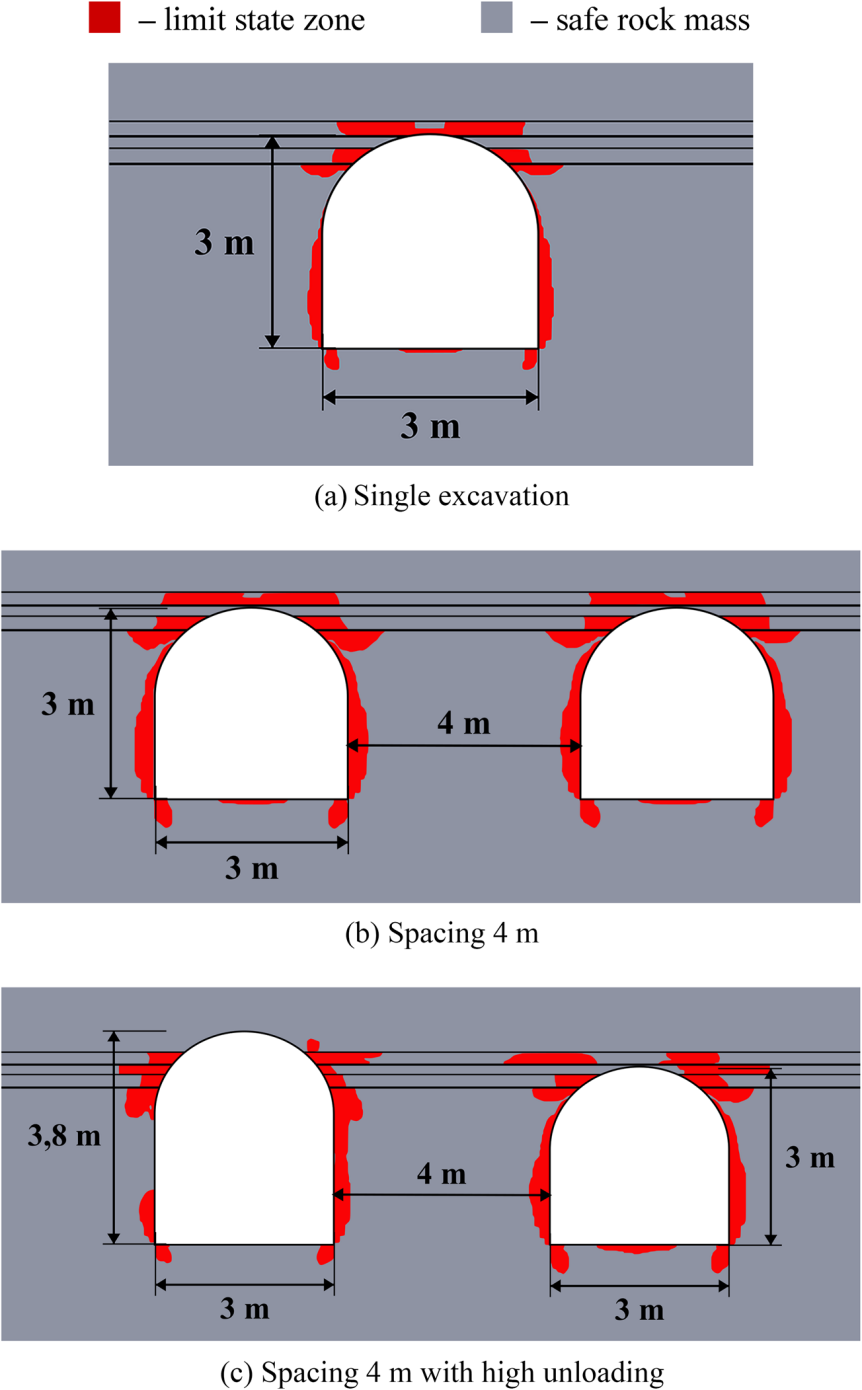

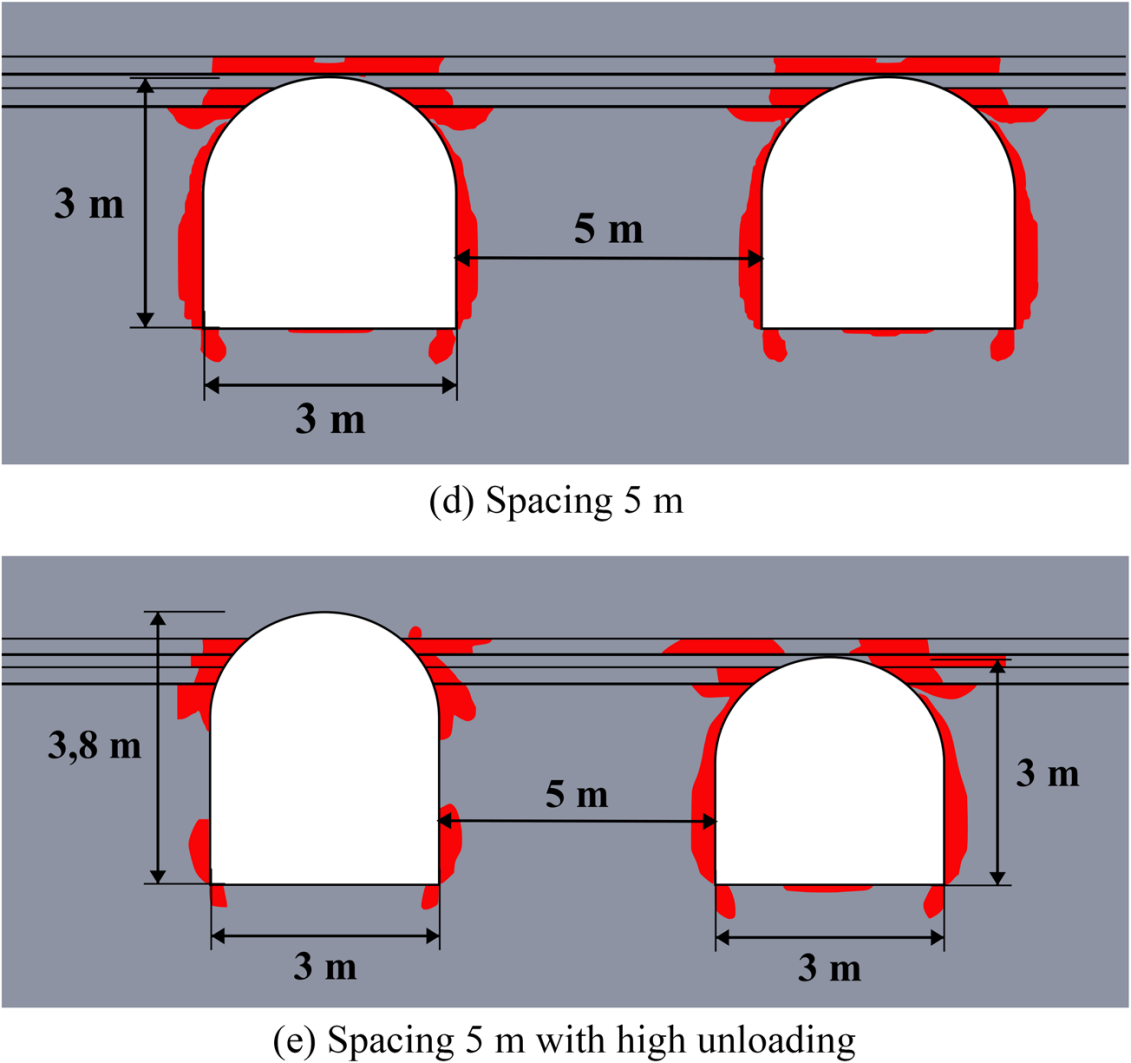
Limit state assessment of considered geotechnical system at the depth of 600 m.

**Fig. 9:**
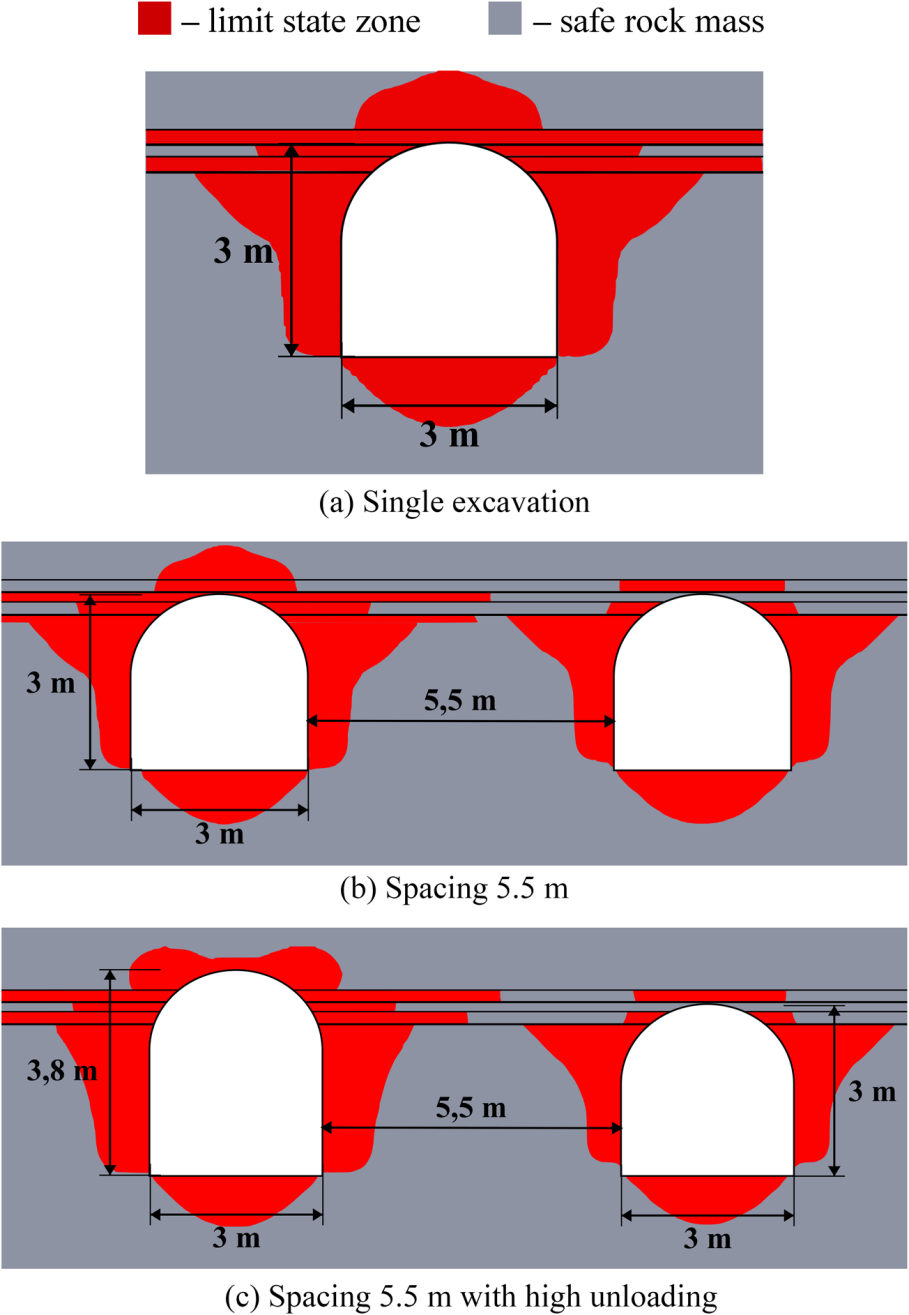

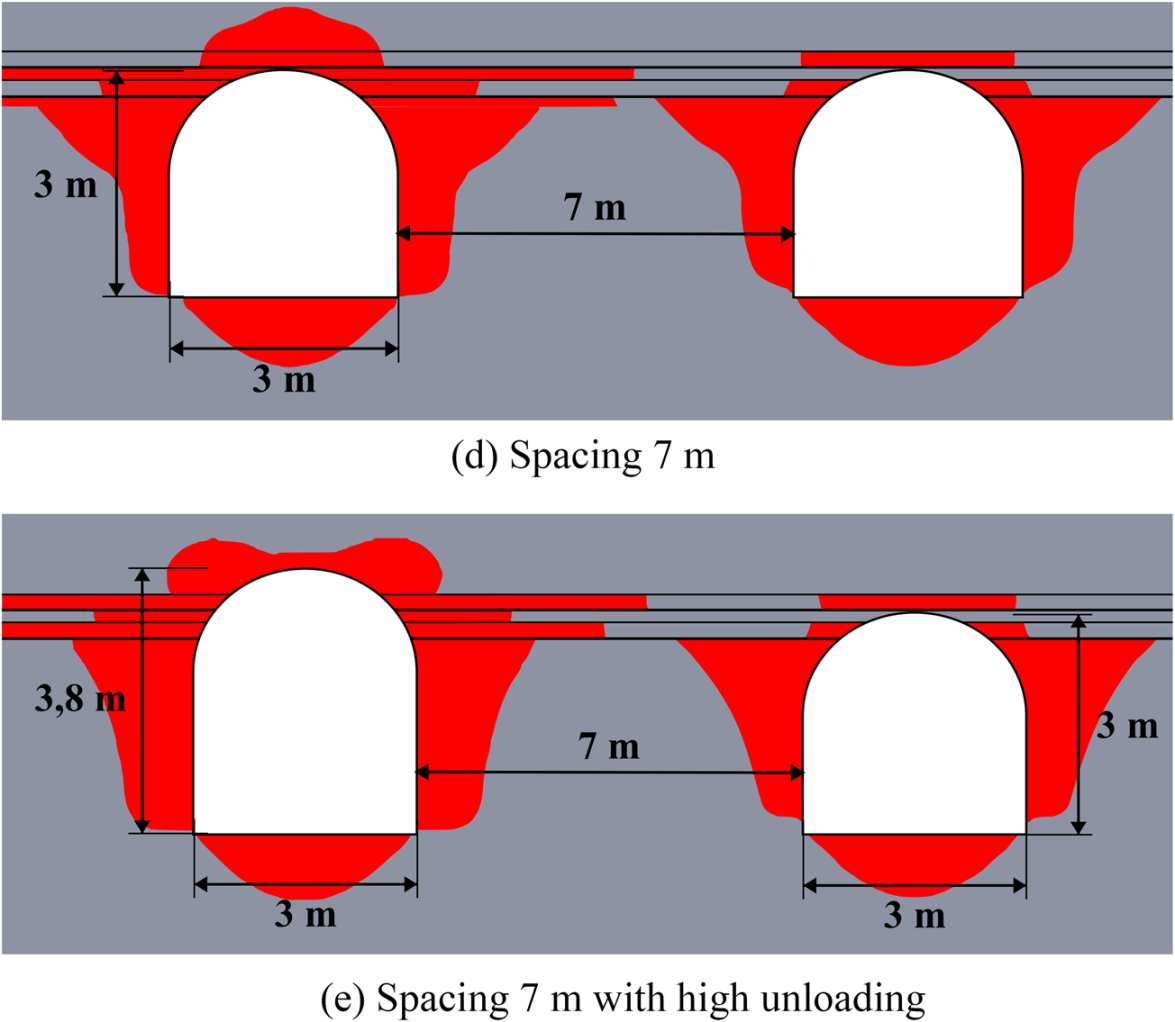
Limit state assessment of considered geotechnical system at the depth of 1200 m.

The results of limit state assessment for considered systems are presented in Figs. 8 and 9 below. Results for moderate depths are presented in Fig. 8, while the results for great depth are presented in Fig. 9. A quantitative comparison of the limit state zones is presented in Table 5.

The results in Fig. 8 show that at moderate depths, the presence of unloading excavations helps to decrease the limit state zones, especially in the roof of the main excavation. This effect stems from a profound redistribution of the stress and strain fields and its characteristics following the creation of the unloading excavation. Consequently, the main excavation is developed within a fundamentally altered regime, characterized by a different magnitude and orientation of principal stresses and strains compared to excavations made in the virgin rock mass. This newly established stress environment significantly influences the limit state and potential failure mechanisms around the main excavation. However, such unloading technique makes the limit state distribution asymmetric, since the limit state zones move to the direction of the unloading excavation. The unloading efficiency can reach up to 45% against up to 100% in the case of the homogeneous rock mass in terms of the limit state zone sizes.

The data of Fig. 9 shows, that at the great depths the unloading is less noticeable (7–37% against 11–40% in homogeneous rock mass), but also significant especially in the roof of the main excavation. The unloading effect for the sides and bottom of the excavations is less than at moderate depths (7–20% vs. 17–26%). However, the key factor here is that the closed-loop limit state zones and potential zonal disintegration here forms only near the unloading excavation, while the main excavation is significantly unloaded due to significant redistribution of the stress-strain state as in the case of moderate depth. In addition, the use of higher unloading excavations is also beneficial, as it moves the limit state zones above the layered strata, thereby increasing the unloading effect and shifting the main stress concentrations away from the main excavation’s outline.

Therefore, in practical applications the fundamental difference in mechanical behavior of the rock mass at moderate and great depths must be taken into account. Thus, at moderate depths the local limit state zones, which are much smaller than the excavation characteristic size can be reinforced and mitigated with the help of traditional safety measures, such as rock bolts. At the same time, larger limit state zones, comparable to the entire dimension of the excavation, which spread deep into the rock mass, occur at great depth, which makes the use of traditional safety measures more complicated and less effective. In this regard, even a moderate decrease of the limit state zone achieved by the unloading excavations is significant for such extremely complex mining environment.

The efficiency of the unloading excavations in the case of the multilayered rock mass is summarized in Table 5 below. This table shows the decrease in the limit state zones for the most optimal placement of the unloading excavation. Note that the distance of 3.5 m for moderate depths and 5.5 m for great depths is considered optimal according to the results of Sect. “Unloading excavations in homogeneous rock mass”. The results of this table also show that in comparison with homogeneous rock mass the unloading effect in multilayered rock mass is less significant, but still noticeable and important.

**Table 5:** Generalized unloading effect values for the multilayered rock mass.

Depth, m	Limit state zone decrease in the excavation side, %	Limit state zone decrease in the excavation roof, %	Limit state zone decrease in the excavation bottom, %	Decrease of the limit state zone total area, %
600	26.2	45	16.6	58.5
1200	20.1	36.5	7.2	29.0

Summarizing the outcomes of Sect. “Analysis of the unloading excavations effectiveness”, the unloading excavations can potentially be used as efficient safety measure for underground structures at all depths in range 300–1200 m in both relatively homogeneous and heterogeneous rock mass.

## Limitations and future work

This section outlines the application scope of the proposed numerical framework, summarizes its inherent limitations, and identifies promising research directions. First, it is crucial to clarify the fundamental scope of the numerical framework presented in this study. Although the ultimate goal of this research is to assess excavation stability and enhance safety in deep mining, the model operates within the theoretical framework of limit state analysis and does not directly simulate dynamic processes such as crack propagation or rock mass collapse. The potential stability assessments and design recommendations are therefore comparative inferences based on the reduction in the calculated extent of limit state zones, rather than direct predictions of failure. Furthermore, to maintain clarity and efficiency in analyzing a vast parameter space, the methodology employs a complex limit state criterion and uses the limit state zones as its primary comparative metric. This approach intentionally prioritizes a systematic comparison of design variants over the presentation of numerous individual stress, strain, or safety factor contour plots, which, while detailed, would be less effective for identifying overarching trends across various simulated scenarios.

While the proposed numerical design framework offers a practical tool for the preliminary assessment of unloading excavations and allows to give general recommendations on their placement, it is essential to acknowledge its limitations, which stem from simplifications made to ensure computational efficiency for extensive parametric analysis. These limitations, in turn, define clear and valuable directions for future research.

The most significant limitation lies in the time-independent nature of the limit state analysis. The FEM provides numerous quasi-static calculations of the stress-strain state and corresponding limit state zones, neglecting the profound influence of time-dependent creep on the evolution of the stress field itself. While this approach is justified for a comparative screening of different geometries under identical conservative assumptions, it cannot capture creep deformation and stress relaxation in time as shown in the validations section. Therefore, a critical next step is the development of a fully coupled, time-dependent visco-elastoplastic model. Such a model would enable a detailed investigation of the time lag between excavations, which is currently omitted from this study, and provide a more physically complete prediction of long-term behavior for many years. The validation results, presented in Sect. “Validation of the results for a single excavation”, are the first step to this direction, but currently they serve to ensure that the displacement field near a single excavation at the depth of 900 m is captured correctly in 1-year perspective, rather than validate the size of the limit state zones directly in all considered cases.

Furthermore, the adoption of 2D plane-strain models represents a simplification of an inherently three-dimensional problem. While sufficient for capturing the fundamental in-plane mechanical interactions between excavations, this approach cannot account for 3D effects such as true stress arching in the out-of-plane direction, and 3D behavior of the pillars. Consequently, for particular practical applications the extension of this framework to 3D is a logical and necessary evolution to ensure the general recommendations are consistent for particular mining cases.

Regarding material modeling, the assumption of perfect bonding between small geological layers ignores potential interface behaviors such as slip or separation. Although this simplification allows for the isolation of the fundamental effects of material contrast, the mechanical response of layered systems is often dominated by interface mechanics^51^. Future work should incorporate cohesive zone models or contact interfaces to investigate how weak boundaries influence stress redistribution and failure mechanisms.

In addition to that, mechanical properties of the rock mass can significantly differ from deposit to deposit, so the presented results should be applied with engineering justification. Thus, for other deposits the optimal location of the unloading excavations should be justified using similar simulations, but with correspondent mechanical properties of rock mass and precise location of geological layers.

Finally, the validation scope of the model remains constrained by the availability of comprehensive field data. The framework was calibrated and validated against a single well-documented case, which, while demonstrating its core capability, limits the confidence in its extrapolation for greater depths. Therefore, a paramount objective for future work is the systematic collection of high-quality field monitoring data from various mining depths and geological conditions. Additional validation cases are required to transform the current practical framework into a robust, predictive tool certified for widespread design use.

In summary, the conclusions in the following section should be regarded as first-order approximations for design guidelines. For specific mining cases, these results should serve as initial recommendations to inform more detailed, site-specific numerical simulations that incorporate time-dependent behavior of rock mass, relevant material properties, 3D geometry, and interfacial phenomena if needed.

## Conclusions and discussion

In this paper the influence of unloading excavations on the limit state of the designed underground structures is studied using extensive series of FEM simulations and comprehensive complex limit state criterion for the assessment of potential critical zones, which occur in the undermined salt rock mass. The use of unloading excavations as a deliberate safety measure for underground openings is a non-classical approach. Unlike conventional methods such as rock bolts or linings, which reinforce the excavation perimeter, unloading excavations require separate, and often costly, mining operations at a distance from the main structures. Despite this disadvantage, such techniques can be indispensable when classical measures are insufficient to ensure safety, particularly in deep mining. The results of the present study demonstrate the efficiency of the unloading excavations on the example of potash mining structures. Based on the results presented above, the following conclusions and recommendations for the design of unloading excavations are established:

1. For small (less than 300 m), moderate (300–900 m) and great (greater than 900 m) depths the mechanical behavior and the limit state zones in the vicinity of excavations significantly differ. At great depths closed-loop limit state rings form in the vicinity of single excavations which can potentially be caused by zonal disintegration effect.
2. The results of numerical studies have conclusively shown that the use of unloading excavations as a standalone protective measure is effective at all depths in the interval 300 to 1200 m, causing 7–100% reduction of the limit state zones. This effect stems not from a mere decrease in individual stress components, but from a fundamental transformation of the local stress-strain state, involving changes in both the magnitude and orientation of the principal stresses and strains throughout the system compared to the virgin rock mass condition.
3. The most efficient distances between main and unloading excavations are: 1.5–6 m for small depths; 3.5–7 m for moderate depths; and 5.5–7 m for great depths. Therefore, the most efficient average distance between excavations lies in the interval from 5 m to 6 m, beyond which the unloading effect diminishes or becomes zero. The most efficient horizontal dimension of the unloading excavations is 3 m. Increasing the horizontal dimensions of the unloading excavation does not significantly enhance the unloading effect, yet it is more costly and complicated to implement.
4. The unloading efficiency for the main excavation’s roof can be enhanced by employing an unloading excavation with an increased height, specifically up to 0.8 m higher than main excavation. This effect is most pronounced at great depths.
5. The use of unloading excavations is efficient in both relatively homogeneous and heterogeneous rock masses. Thus, in case of multilayered rock mass, the unloading can reach 7–45%, which is significant especially for great depth.
6. The unloading effect at great depths, while less pronounced than at moderate depths, is still significant (up to 37–39%). A key advantage here is that the closed limit state zones form only in the vicinity of unloading excavation, but not at the outline of the main excavation. However, the optimal parameters for this measure at great depths are different from those at moderate depths.
7. The use of unloading excavation primarily helps to decrease the shear stress in the rock mass at the excavations outline. The decrease of compressive and tensile stress can reach 8–10% due to the unloading effect, which is noticeable, but not decisive.

The combined application of the proposed computational methodology and the unloading excavation technique can potentially enhance mining safety by decreasing the limit state zones near underground openings. Furthermore, unloading excavations can be integrated with traditional support measures, such as rock bolts and linings, to create a more robust safety system. The conclusions above should be applied considering the limitations described in the previous section. Establishing the quantitative parameters for the vast majority of possible cases falls outside the scope of this study and requires further research. However, modern automated parameter identification systems can serve to fulfill this gap^52^.

## Data Availability

All data generated or analysed during this study is included in this published article and corresponding references.
